# Heat stress reprograms herbivory-induced defense responses in potato plants

**DOI:** 10.1186/s12870-024-05404-x

**Published:** 2024-07-17

**Authors:** Jian Zhong, Jinyi Zhang, Yadong Zhang, Yang Ge, Wenjing He, Chengjuan Liang, Yulin Gao, Zengrong Zhu, Ricardo A. R. Machado, Wenwu Zhou

**Affiliations:** 1 State Key Laboratory of Rice Biology and Breeding, Ministry of Agricultural and Rural Affairs Key Laboratory of Molecular Biology of Crop Pathogens and Insect Pests, Institute of Insect Sciences, Zhejiang University, Hangzhou, 310058 China; 2 Hainan Institute, Zhejiang University, Sanya, 572000 China; 3 State Key Laboratory for Biology of Plant Disease and Insect Pests, Institute of Plant Protection, Chinese Academy of Agricultural Sciences, Beijing, 100081 China; 4 Experimental Biology Research Group, Institute of Biology, University of Neuchatel, Neuchatel, 2000 Switzerland

**Keywords:** High temperature, *Solanum tuberosum*, *Phthorimaea operculella*, Plant resistance to insect herbivores, Phylotranscriptomic analysis, Metabolic reprogram

## Abstract

Climate change is predicted to increase the occurrence of extreme weather events such as heatwaves, which may thereby impact the outcome of plant-herbivore interactions. While elevated temperature is known to directly affect herbivore growth, it remains largely unclear if it indirectly influences herbivore performance by affecting the host plant they feed on. In this study, we investigated how transient exposure to high temperature influences plant herbivory-induced defenses at the transcript and metabolic level. To this end, we studied the interaction between potato (*Solanum tuberosum*) plants and the larvae of the potato tuber moth (*Phthorimaea operculella*) under different temperature regimes. We found that *P. operculella* larvae grew heavier on leaves co-stressed by high temperature and insect herbivory than on leaves pre-stressed by herbivory alone. We also observed that high temperature treatments altered phylotranscriptomic patterns upon herbivory, which changed from an evolutionary hourglass pattern, in which transcriptomic responses at early and late time points after elicitation are more variable than the ones in the middle, to a vase pattern. Specifically, transcripts of many herbivory-induced genes in the early and late defense stage were suppressed by HT treatment, whereas those in the intermediate stage peaked earlier. Additionally, we observed that high temperature impaired the induction of jasmonates and defense compounds upon herbivory. Moreover, using jasmonate-reduced (JA-reduced, *irAOC*) and -elevated (JA-Ile-elevated, *irCYP94B3s*) potato plants, we showed that high temperature suppresses JA signaling mediated plant-induced defense to herbivore attack. Thus, our study provides evidences on how temperature reprograms plant-induced defense to herbivores.

**Supplementary Information:**

The online version contains supplementary material available at 10.1186/s12870-024-05404-x.

## Introduction

Plants have evolved sophisticated mechanisms to counteract insect herbivore attacks and decrease the fitness penalties imposed by the damage they cause [1]. How abiotic stress modulates plant herbivory-induced defensive responses has been subject of intense research over the last years [2, 3]. How carbon dioxide, drought, temperature, and radiation, for instance, influence plant herbivory-induced defenses has emerged as an interesting research topic and has been investigated in several different plant systems [4–6]. More recently, the impact of climate change on different aspects of plants, including the interaction with herbivores has attracted considerable attention [5, 7, 8]. Under a climate change scenario, the occurrence of heat waves is predicted to increase [9]. How heat waves influence plant herbivory-induced defensive responses to insect attack in potato remains poorly investigated [10, 11].

Heat waves and high temperatures may directly influence arthropod herbivores by impacting their development, survival, and fecundity, but also indirectly by affecting their performance via plant-mediated effects [12, 13]. For instance, heat waves can influence plant-induced defensive responses to herbivore attack [14]. Temperature-sensitive plants experience greater herbivory damage in warmer years [15]. Understanding the direct and indirect effects of heat waves on herbivore performance is therefore of crucial relevance if we are to develop strategies to mitigate the potential negative consequences of climate change in agriculture.

Plant herbivory-induced defense responses are a dynamic process divided into three distinct stages: early, intermediate, and late [16]. In the early stage, plants perceive herbivores by detecting herbivore-specific patterns, such as wounding damages, elicitors, and activate early defense signals such as electrical signals, hydraulic waves, calcium (Ca^2+^), and reactive oxygen species (ROS) [17, 18]. These early signaling events trigger downstream signaling events including the induction of phytohormonal pathways [19, 20]. Jasmonic acid (JA) and salicylic acid (SA) are the main hormones in the defense against herbivores, but other hormones such as abscisic acid (ABA) and auxins (IAA) are also involved [21, 22]. In the intermediate stage, the modulation of plant primary metabolism occurs, including altered photosynthesis and sink/source relations [23]. In most cases, this modulation is accompanied by the subsequent synthesis of specialized metabolites [24, 25]. In the later stage, the production of defensive compounds occurs, which triggers plant defensive status and resistances against herbivore attack [26]. Remarkably, these three stages can also be captured at the transcriptomic level. Transcript levels of defense-signaling genes are altered first, then the transcript levels of primary metabolism-related genes, and lastly the transcript levels of specialized metabolism-related genes [27].

Transcriptomic studies on plant’s response to herbivores show that the time-dependent activation for the three functional gene groups (defense-signaling, primary metabolism, and specialized metabolism) is common in many plants [28, 29]. This provides evidence on how plants optimize the allocation of photosynthetic resources in a sequential manner. Meanwhile, due to the selective pressure that herbivores inflict on plants in nature [30], these three gene groups also show unique evolutionary characteristics. Using phylotranscriptomic methods, the induced transcriptome age index (iTAI) and induced transcriptome divergence index (iTDI) of these three gene groups have been analyzed, and their evolutionary properties showed an ‘hourglass (high-low-high) pattern’ trend in *Nicotiana attenuata*: the primary metabolism gene group at middle time points has strong evolutionary constraints, whereas the defense-signaling and specialized metabolism at early and late time points have high divergent selection or relaxed purifying selection [27]. Meanwhile, numerous studies have also found that plants have evolved distinct functional gene groups and transcriptomic responses to cope with abiotic stresses [31, 32]. It remains largely unclear how the activation of herbivore defense response related functional gene groups is affected by high temperatures and heat waves in plants.

Extreme temperature also significantly impacts plant metabolism, and in turn, modulates plant-herbivore interactions [33, 34]. For instance, the concentration of many specialized metabolites, including flavonoids, phenolics, and terpenoids, are altered in plants grown under high temperatures, which subsequently affects the performance of herbivores on plants [35, 36]. This is partially attributed to high temperature-driven modification of the activity of plant enzymes responsible for the biosynthesis of these metabolites [37, 38]. Apart from endogenous metabolites, the emission of volatile organic compounds is also modulated by temperature. In potato, a cool-weather crop, for instance, high temperature enhances the emission of one key volatile organic compound (ß-caryophyllene) from herbivory-induced leaves, which reduces the attractiveness of this plant to *P*. *operculella* adult moths but enhances its recruitment to the parasitoid wasp *Trichogramma chilonis* of this pest [39], indicating that temperature can affect potato’s induced defenses against these insects.

In this study, we investigated if heat waves (transient increase in temperature) modulate the herbivory-induced defensive responses of potato plants to the attack of *P*. *operculella*. We tested the hypothesis that high temperature constrains potato plant-induced defenses against the attack of *P*. *operculella* by reshaping phylotranscriptomic patterns of herbivory-responsive genes, influencing the accumulation patterns of jasmonate-associated, primary metabolism- and specialized metabolism-related genes, and by modulating the production of primary and specialized metabolites. To this end, we conducted a series of experiments including transcript, phytohormone, and metabolite measurements, and insect performance analysis. Moreover, we conducted phylotranscriptomic, transcript functional annotation followed by targeted gene expression profiling, and weighted gene co-expression network analysis (WGCNA) to investigate how temperature modulates herbivory-induced responses in potato, thereby providing important insights into the potential consequences of climate change in plant-herbivore interactions.

## Materials and methods

### Plants and planting conditions

Two *Solanum tuberosum* varieties were used in this study: Solanum group Tuberosum RH89-039-16 (RH) and *Solanum tuberosum* cultivar E-Potato 3 (E3). Potato plants were generated via tissue culture on MS medium (4.43 g L^− 1^ Murashige and Skoog medium, 30 g L^− 1^ sucrose and 8 g L^− 1^ agar) in a climate chamber (16/8 h light/dark; 22 ± 1 °C; 65% relative humidity). Three-week-old plantlets were then planted into 1 L pots with soil and grown in a greenhouse (16/8 h light/dark; 22 ± 1 °C/16 ± 1 °C; 65% relative humidity) with a light intensity of 600–800 µmol m^− 2^ s^− 1^ (600 W, Lucagrow, Hungary).

### Insects and insect rearing

The adults and larvae of *P*. *operculella* used in this study were initially collected from a potato field in Yunnan Province, China. A laboratory colony was established from these insects and has been maintained on potatoes in a climate chamber (16/8 h light/dark; 26 ± 1 °C; 60–70% relative humidity) for more than 30 generations.

### Pre-herbivory treatment and heat wave induction

Simulated herbivory treatments (wounding + OS_PTM_) were used instead of real herbivory to standardize damage levels and induction timing [40, 41]. *P*. *operculella* oral secretions (OS_PTM_) were collected from 3rd -4th instar larvae. Larvae were starved for 6 h and then fed with potato leaves for 12 h. OS_PTM_ was collected using a fine capillary pipette (internal diameter: 0.5 mm) while being maintained on ice. Prior to simulated herbivory treatments, twenty-day-old potato plants were transferred into two growth chambers at either high temperature (HT; 16/8 h light/dark; 35 ± 1 °C/28 ± 1 °C; 65% relative humidity) or control temperature (CT; 16/8 h light/dark; 22 ± 1 °C/16 ± 1 °C; 65% relative humidity). Plants were kept under the different temperature regimes for 3 h (between 6am and 9am) before starting the simulated herbivory treatments. For the simulated herbivory treatments, the second fully-expanded young leaves of the plants were subjected to *P*. *operculella* simulated herbivory. Actual herbivory caused by *P*. *operculella* includes both mechanical wounding and the introduction of chemical molecules present in the insect oral secretions (OS_PTM_) to the wounds [40]. To simulate *P*. *operculella* herbivory, mechanically wounded plants were treated with 20 µL 1:5 diluted OS_PTM_ [42]. Untreated plants were used as the controls. Mechanical wounding treatments were made by rolling a pattern-tracing wheel on the leaves. Each leaf received six rows of evenly distributed tracing wheel damage on each side of the mid-vein. Leaf samples were harvested at 0, 0.5, 1, 5 and 11 h post herbivory treatment, frozen, subsequently macerated in liquid nitrogen at − 80 °C till use for transcript and metabolite quantifications (Fig. 2A). The time points selected for sample collection were based on herbivory-induced dataset analysis in Durrant’s study [27].

**Fig. 1:**
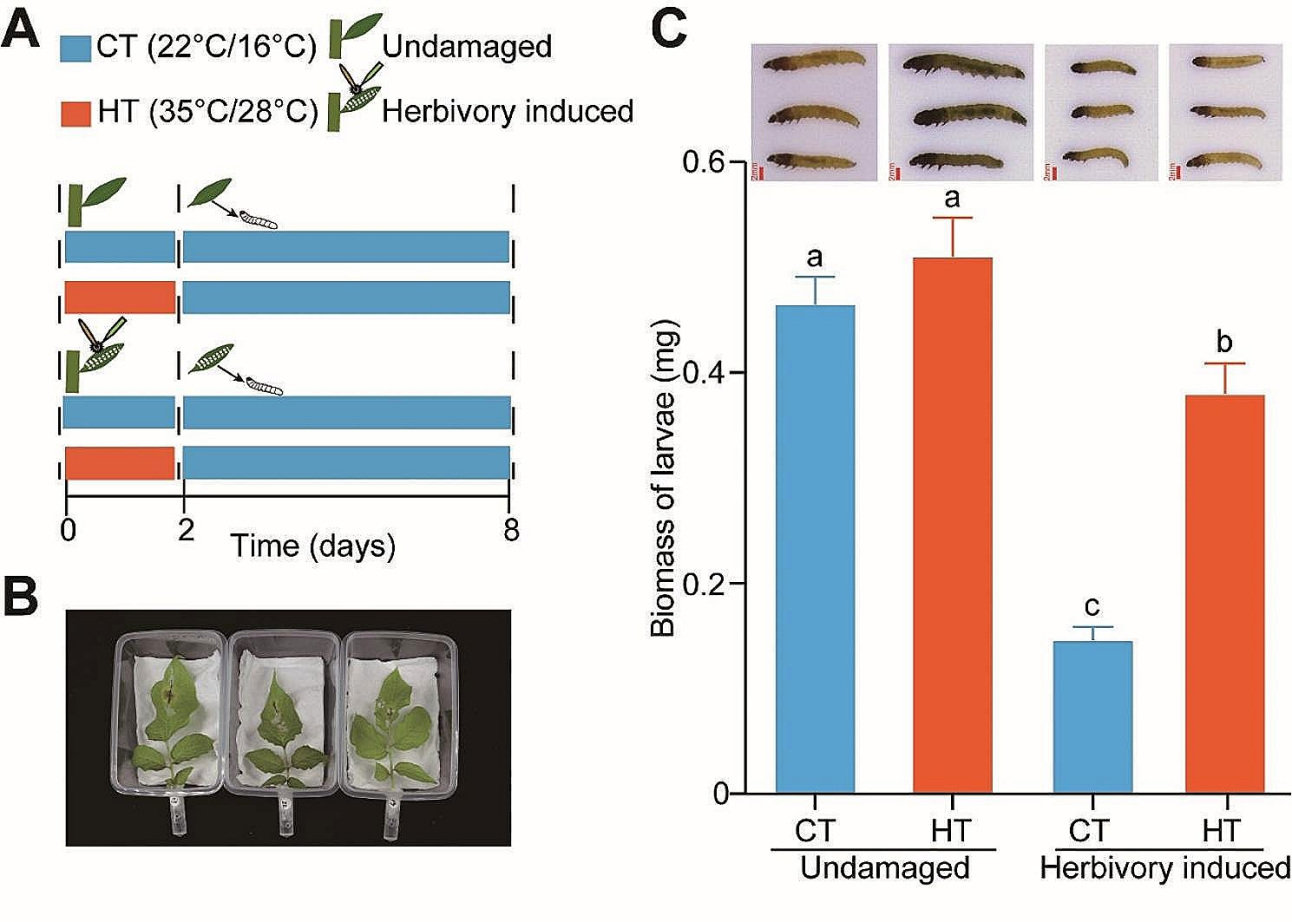
*Phthorimaea operculella* larvae grew heavier on leaves co-stressed by high temperature and insect herbivory than on leaves pre-stressed by herbivory alone **(A**,** B)** Experiment setup of insect bioassays. Twenty-day-old potato plants grown in CT condition were transferred to CT or HT treatment chambers, then they were pre-treated with *P. operculella* simulated herbivory for two days, and then their leaves were cut off and used to fed larvae for six days at CT regime in plastic transparent boxes. **(C)** Larval weights on leaves (*n* = 30). Different letters on the top of the columns indicate differences at *P* < 0.05. Error bars represent the mean ± standard error (SE) for each

### Insect bioassays on detached leaves

To differentiate the direct effect of high temperature on *P*. *operculella* larvae development from the effect of high temperature on potato plant defense response to larvae. We evaluated insect performance on leaves co-stressed with insect herbivory and high temperature, pre-stressed with insect herbivory alone, and pre-stressed with high temperature alone. Plants grown in control temperature without insect herbivory were used as control. The performance of insect herbivores was measured on detached leaves following the methods by Xu et al. [43]. Second fully-expanded leaves of twenty-day-old potato plants pre-exposed to HT or CT for 3 h were treated with simulated herbivory, and the undamaged plants were used as control (Fig. 1A). The plants were then continuously maintained under HT or CT for two days. After that, leaflets were harvested and transferred into plastic transparent boxes (11 × 8 × 4 cm) to feed larvae for six days. Boxes were kept in CT growth chambers (16/8 h light/dark; 22 ± 1 °C/16 ± 1 °C; 65% relative humidity). To prevent desiccation, the petioles were wrapped in wet cotton wool (Fig. 1B). Two neonate larvae were then released in each box and allowed to feed for six days. After this period, all larvae were collected (*n* = 30), weighed, and photographed.

**Fig. 2:**
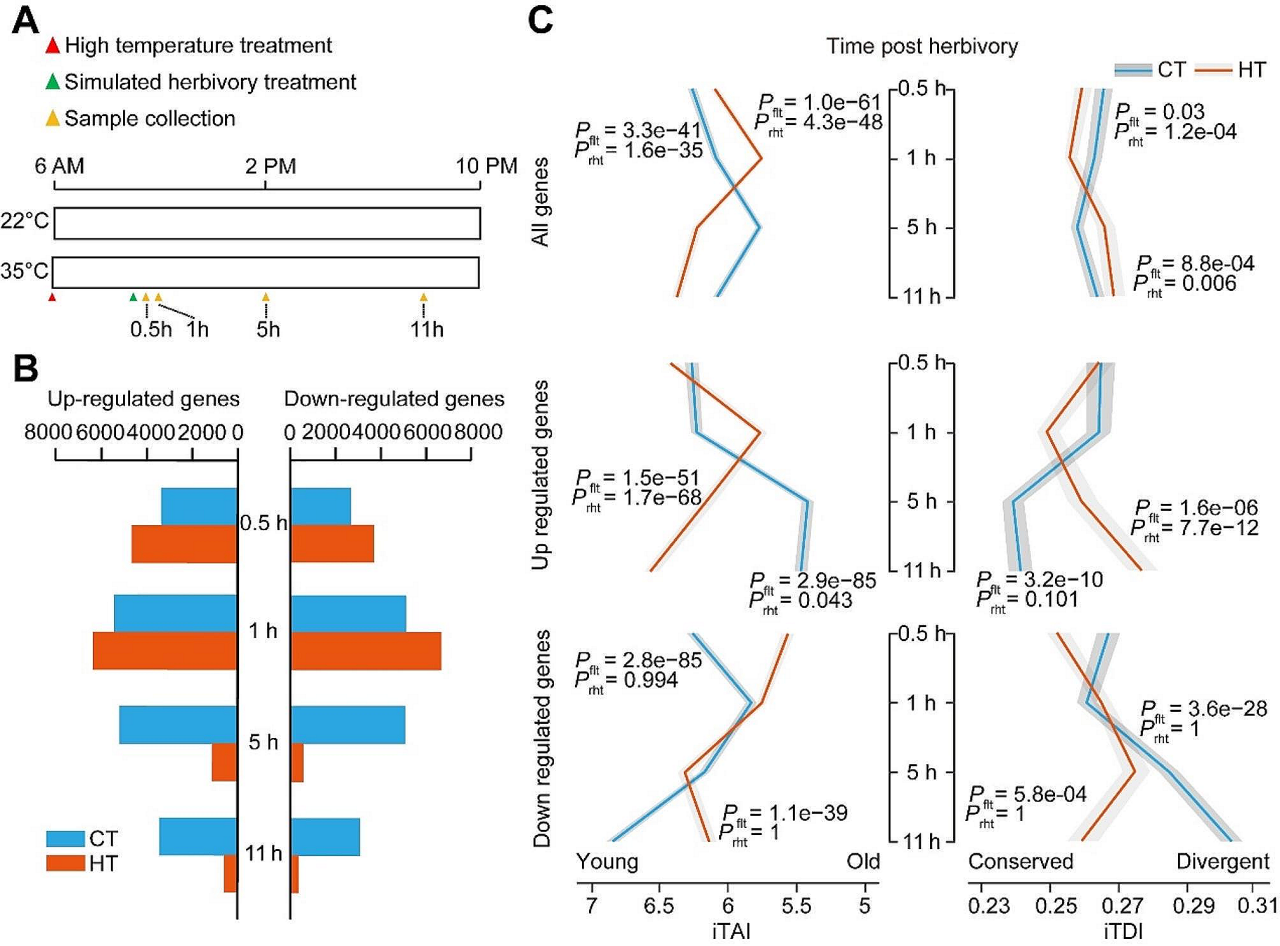
Herbivory-induced transcriptomic responses and phylotranscriptomic pattern were affected by high temperature. **(A)** Experimental setup of RNA-Seq treatments and sampling. Potato plants were kept in control temperature (CT; 22 °C) or high temperature (HT; 35 °C) conditions at 6 am, and then treated with *P. operculella* simulated herbivory at 9 am; after that leave samples were collected at 0.5 h, 1 h, 5 h, 11 h post herbivory. **(B)** Number of herbivory-responsive up- and down-regulated genes at CT/HT at different time points. **(C)** iTAI and ITDI are shown in the left and right, respectively. Each row from the top to the bottom designate mean indices calculated on all herbivory-responsive genes, up- and down-regulated genes, respectively. The grey ribbons represent standard deviation (SD). *P*_flt_ and *P*_rht_ suggest the *P*-values from a flat line and reductive hourglass tests, respectively. *P*_flt_ < 0.05 indicates the pattern is significantly different from a flat line and *P*_rht_ < 0.05 indicates it follows an hourglass pattern

### Plasmid construction and potato transformation

To impair the production of jasmonates in potato plants, we silenced the allene oxide cyclase (*AOC*) gene that code for a crucial enzyme for jasmonate biosynthesis [44]. Briefly, a 250 bp fragment from the *AOC* coding sequence and its reverse sequence were combined to two restriction enzyme cutting sites (*Xho*I and *Xba*I) of the pHELLSGATE8 vector using homologous recombination technology. The construct was then introduced into *Agrobacterium tumefaciens* strain GV3101, which was then transformed into E3 cultivar as previously described [45]. Gene silencing efficiency was tested in several different transgenic lines (Fig. S4). The sequences of the primers using for *AOC* gene clone and RNA interference are shown in Table S1. The jasmonate-elevated E3-potato transgenic lines, *irStCYP94B3s*, in which the three JA-isoleucine (JA-Ile) hydroxylases were all simultaneously silenced, were also used in our research [46].

### RNA-seq

To understand if and how temperature affects evolutionary properties of herbivory-induced genes in potato plants, we conducted RNA-seq and phylotranscriptomic analyses in plants challenged by simulated herbivory under two temperature regimes: HT and CT. Total RNA was extracted with a plant RNA extraction kit (Zhejiang Easy-Do Biotech CO., LTD). The purity and concentration of RNA were quantified using a NanoDrop spectrophotometer (Thermo Scientific). For each sample, 1 µg of high-quality RNA was used for RNA-seq, where strand-specific sequencing libraries were generated using NEBNext^®^ UltraTM RNA Library Prep Kit for Illumina^®^ (NEB, USA) and sequenced with Hiseq-PE150 platform.

The raw data in FASTQ format was processed using Fastp v0.20.0 [47]. The resulting data were then aligned to potato genome (PGSC_DM_v4.03) with STAR in a 2-pass mode [48]. All RNA-Seq reads data were deposited in Short Read Archive (SRA) at the NCBI database (Accession: PRJNA903553). Aligned reads were transferred to StringTie to generate assembled transcripts; the transcripts were then merged into a single unified transcriptome assembly with TACO v0.6.2 [49]. Gffcompare v0.11.2 [50] was used to evaluate and compare the resulting unified transcriptome assembly with the known genome annotation via different transcript classification codes; transcripts with “u” were selected for novel transcripts’ filtering (splice reads > 10 in all samples). Salmon v1.4.0 [51] was used to compute transcript per million (TPM) accumulation for each transcript and only transcripts with a TPM of ≥ 1 in at least one sample were used for downstream differential expression analysis. The novel transcripts were subjected to TransDecoder v5.5.0 [52] to identify putative longest open reading frame (ORF) [53]. Differential expression analysis was performed by the R package DESeq2 [54]. Genes were considered significantly differentially expressed if their |log_2_fold-change| ≥1 and the adjusted *P*-value < 0.05.

### Phylostratigraphy and *K*_a_*/K*_s_ ratios

Phylostratigraphic analyses were carried out using a custom Perl script [55]. Briefly, protein sequences were searched in the non-redundant (nr) NCBI protein database (downloaded in 2019) using the BLASTP algorithm at an e-value cutoff of 1e-05. The BLASTP results were filtered to exclude viruses and sequences of non-cellular organisms. Subsequently, all genes were assigned to 13 phylogenetic ranks (PS, phylostrata), starting from the origin of ‘cellular organism’ and ending at *S. tuberosum* following a custom Python script in Durrant’s study [27].

The *K*_a_*/K*_s_ ratio for each gene was obtained from the PAML v4.9 package [56] by performing pairwise sequence comparisons between two closely relared species: potato (*S. tuberosum*) and tomato (*S. lycopersicum*). The *K*_a_*/K*_s_ ratio was calculated from aligned protein coding sequences. Genes with a K_s_ value > 0.05 and < 1 were retained for future analysis [27].

### Transcriptome induction indices

To investigate how HT influences the evolutionary properties of the genes induced by *P*. *operculella* simulated herbivory, we calculated two transcriptomic induction indices: the induced transcriptome age index (iTAI), which estimates gene age (PS) weighted by gene induction (log_2_fold-change), and the transcriptome divergence index (iTDI), which estimates sequence divergence (*Ka/Ks*) weighted by gene induction (log_2_fold-change). iTAI and iTDI of each gene induction stage was calculated via myTAI package, respectively [57]. The iTAI and iTDI are defined as:

$$\:{\mathbf{i}\mathbf{T}\mathbf{A}\mathbf{I}}_{\mathbf{t}}=\frac{{\sum\:}_{\mathbf{i}=1}^{\mathbf{n}}{\mathbf{P}\mathbf{S}}_{\mathbf{i}}\left|{\mathbf{F}\mathbf{C}}_{\mathbf{i}\mathbf{t}}\right|}{{\sum\:}_{\mathbf{i}=1}^{\mathbf{n}}\left|{\mathbf{F}\mathbf{C}}_{\mathbf{i}\mathbf{t}}\right|}$$

$$\:{\mathbf{i}\mathbf{T}\mathbf{D}\mathbf{I}}_{\mathbf{t}}=\frac{{\sum\:}_{\mathbf{i}=1}^{\mathbf{n}}\left(\frac{{\varvec{K}}_{\mathbf{a}\mathbf{i}}}{{\varvec{K}}_{\mathbf{s}\mathbf{i}}}\right)\left|{\mathbf{F}\mathbf{C}}_{\mathbf{i}\mathbf{t}}\right|}{{\sum\:}_{\mathbf{i}=1}^{\mathbf{n}}\left|{\mathbf{F}\mathbf{C}}_{\mathbf{i}\mathbf{t}}\right|}$$

where t is a time point, n is the number of genes for analyzing, PS_i_ is the PS of gene I, |FC_it_| is the absolute log_2_fold-change value of gene i at time point t, *K*_a.i._*/K*_si_ is the *K*_a_*/K*_s_ value of gene i. High iTAI_t_ indicates that the transcriptome induced at time point t is evolutionarily young, whereas low iTAI_t_ suggests ancient. High iTDI_t_ indicates that the transcriptome induced at time point t is more divergent, whereas low iTDI_t_ represents a more conserved transcriptome. To characterize early, intermediate and late transcriptomic responses, transcript levels were evaluated at four time points after simulated herbivory: 0.5 h and 1 h (early), 5 h (intermediate), and 11 h (late).

### Methyl jasmonate (MeJA) treatment

Exogenous applications of MeJA is widely used to increase jasmonate levels and mimic herbivore attack in plants. MeJA (Solarbio, China) was dissolved in liquefied lanolin paste (7.5 mg/ml) in a warm (50 °C) water bath. About 0.02 ml of lanolin paste containing MeJA were then smeared on the base of leaf blades of the second fully-expanded young leaves of twenty-day-old potato plants [58]. Control plants were treated similarly with pure lanolin paste. After designated time, the tip of lead blade which was not smeared with MeJA was cut for detached leaf bioassay and metabolite analysis. Untreated plants were left intact.

### Weighted gene co-expression network analysis (WGCNA) and gene set identification

Weighted gene co-expression network analysis was carried out using the “WGCNA” package in R 3.6.0 [59]. The “sva” package and the “ComBat” function was used to reduce the effects of background expression differences between the two cultivars RH and E3 [60]. RNA-seq samples were used to calculate the soft connectivity, after that the top 5000 connected genes were selected for module construction with parameters ‘softPower = 16, and minModuleSize = 300’; the module eigengenes (MEs) were then calculated and clustered by threshold of 0.15, therefore the modules whose eigengenes were correlated above 0.85 were merged.

To identify which module showed the highest correlation with herbivory-induced jasmonic acid (JA) accumulation, module–trait relationships were estimated using the correlation between MEs and JA. The corresponding gene information for the most correlated module with membership greater than 0.75 was defined as an early defense signaling (or early jasmonate-associate) gene set [61]. In order to acquire primary and specialized metabolism gene sets, protein sequences of *S. tuberosum* were subjected to eggnog-mapper v2 to get functional annotations per query including EC numbers in Table S2 [62], which were combined with those deposited in PMN database (http://ftp://ftp.plantcyc.org/pmn/Pathways/Data_dumps/PMN15_January2021/pathways/potato_pathways.20210325.txt). The EC-annotated genes were mapped to either primary metabolism pathway or specialized metabolism pathway following the study by Chae et al. [63].

### Gene expression analysis

To further explore how the transcript levels of three identified herbivory-induced gene sets (the early defense signaling molecule jasmonate-associated, primary metabolism-, and specialized metabolism-related genes) changed under different temperature regimes, the median of gene transcript accumulation was calculated and transformed by row scaled (z-score) log_2_(median + 1). Lines were then drawn by concatenating the mean of the median data of replicates between two adjacent time points, where we referred to the principle of the ‘PlotGroups’ function from the maSigPro package [64]. Heatmaps were plotted with row-scaled (z-score) log_2_(TPM + 1) transformed expression data using the R package “pheatmap” [65].

### Quantitative real-time PCR (qRT-PCR) validation of gene transcript accumulation

The relative of gene expression levels were calculated based on standard curves. Primers used for qRT-PCR were designed using the primer blast function of NCBI (https://www.ncbi.nlm.nih.gov/tools/primer-blast/). All primer sequences are listed in Table S1. *Ef3d* was used as the housekeeping gene [66]. 1 µg RNA was treated with gDNA Remover kit (Toyobo, Osaka, Japan) to remove genomic DNA and subsequently reverse transcribed into cDNA using Toyobo qRT-PCR kit (Toyobo). Quantitative real-time PCR was carried out in 96-well plates using a CFX96 real-time PCR system (Biorad, USA), with SYBR green fluorescent dye, according to the manufacturer’s instructions (Toyobo). The cycling parameters for all genes were the following: 1 cycle at 95 °C for 3 min, 39 cycles at 95 °C for 10 s, and at 60 °C for 30 s, followed by a melting curve analysis at 95 °C for 5s, at 65 °C for 60s, and at 95 °C for 15s.

### Phytohormone measurements

To quantify phytohormones, 100 mg of macerated leaf samples were extracted with 0.5 mL 80% methanol. 1 µL internal standards (10 ng µL^− 1^, D_6_JA, D_4_SA, D_6_JA-Ile and D_6_ABA) were added per sample. The samples were then vortexed and kept at 4 °C overnight. After this, all samples were dried with a constant flow of nitrogen gas. Then, samples were reconstituted by adding 200 µL ethyl acetate and vortexing for 5 min. The organic layer was then transferred into a new microtube and dried again with a constant flow of nitrogen gas. All samples were then dissolved in 100 µL 50% methanol, vortexed for 5 min, and centrifuged at 10,000 rpm for 5 min. The samples were finally disposed into glass vials (Agilent, USA) and analyzed by HPLC-MS (6400 Series Triple Quadrupole LC/MS Systems, Agilent, USA) [42].

### Soluble sugar measurements

Soluble sugar measurements were performed as described by Xiang and Tang [67], with some minor modifications, i.e.: 0.5 mL of extraction buffer were used instead of 4 mL. Briefly, 50 mg of macerated leaf samples were extracted with 0.5 mL cooled methanol: chloroform: water (12:5:3 v/v/v). An internal standard (1 µL of 2000 ng µL^− 1^ ribitol) was added to each sample. The samples were then vortexed thoroughly. After this, 500 µL water were added to all samples, and all samples were centrifuged at 10,000 rpm for 10 min. Supernatants were then transferred to a 2 mL scintillation vial, and lyophilized using a freeze dryer (Ningbo Scientz Biotechnology CO., LTD). All samples were then supplemented with 25 µL hydroxyamine hydrochloride (100 mg mL^− 1^ in 1-Methylimidazole) and incubated in a water bath at 80 °C for 5 min. Then, 25 µL of acetic anhydride were added to the samples. Samples were then kept at room temperature for 5 min for acetylation. After this, 200 µL of chloroform were added to the samples. Samples were then washed with 500 µL ddH_2_O for 3–4 times. Lastly, 100 mg anhydrous sodium sulfate (Sinopharm Chemical Reagent Co., Ltd) were added to the samples. Soluble sugar levels were then determined by gas chromatography coupled with mass spectrometry (GC-MS) (6890 N gas chromatograph by Agilent with a GCT Premier mass spectrometer by Waters) using a SIM mode.

### Steroidal glycoalkaloids (SGAs) measurements

α–solanine and α–chaconine are the major SGAs in potatoes [68]. SGAs measurements were carried out as described, with some modifications [69]. Specifically, we proportionally reduced the volume of the extract reagent and the samples. The extract reagent was changed from 5% aqueous acetic acid to 10% methanol solution of acetic acid. In brief, 50 mg of macerated leaf samples were extracted with 5 mL cooled acetic acid: methanol (1:10 v/v). Then, all samples were vortexed for 30 min. The homogenates were then centrifuged (5000 rpm, 10 min, 4 °C) and the residue re-extracted three times using the same procedure. Lastly, the extracts were combined and all samples were filtered through 0.22 μm organic membranes. SGAs were profiled by HPLC-MS (6400 Series Triple Quadrupole LC/MS Systems, Agilent, USA).

### Trypsin proteinase inhibitor (TPI) measurements

TPI measurements were performed as described by van Dam et al. [70]. Briefly, 100 mg of macerated leaf samples were extracted with 0.3 mL cooled extraction buffer (0.1 M Tris-C1, pH 7.6, 5% polyvinylpolypyrrolidone, 2 mg mL^− 1^ phenylthiourea, 5 mg mL^− 1^ diethyldithiocarbamate, 0.05 M Na_2_EDTA). Then, all the samples were vortexed for 5 min and centrifuged (12,000 rpm, 20 min, 4 °C). Supernatants were transferred to ELISA plates and analyzed to quantify total protein levels. TPI activity was then evaluated in the extracts using the radial diffusion method [70].

### Statistical analyses

The insect bioassay data on plants and detached leaves were analyzed using one-way analysis of variance (ANOVA) and Student’s *t*-tests with default parameters to test the significance of differences among treatment groups. Gene expression data for each stage post herbivory at HT/CT were analyzed using a *t*-test. Data on phytohormones, soluble sugar, SGAs, and TPI among treatment groups were analyzed using Student’s *t*-tests at each time point. Differences were considered statistically significant when *P*-values were lower than 0.05. The data were presented as means ± standard errors. To test whether the iTAI and iTDI values were significantly different from a flat line and consistent with an hourglass pattern, the permutation tests described in Drost et al. [71] were used (10,000 permutations and 100 runs) with *P*_flt_ < 0.05 and *P*_rht_ < 0.05. For the correlation coefficient with JA in WGCNA analysis, differences across modules were determined using Wilcoxon-Mann-Whitney test.

## Results

### Temperature reduces plant-induced defense to *P*. *operculella* herbivory

A detached leaf bioassay was performed on RH cultivar (Fig. 1A). Insect grew similarly when fed on either undamaged leaves of plants pre-exposed to HT or on undamaged leaves of plants pre-exposed to CT. Interestingly, when insects fed on leaves previously subjected to simulated herbivory, they grew less compared to when insects fed on undamaged leaves, while temperature modulated this herbivory-induced effect from the results that insects grew better when fed on leaves co-stressed by high temperature and insect herbivory than on those pre-stressed by herbivory alone (Fig. 1C). Collectively, these results suggest that HT influences plant-induced defenses to herbivore attack.

### Temperature shapes phylotranscriptomic patterns of herbivory-responsive genes in potato plants

The herbivory-induced transcriptomic responses and phylotranscriptomic pattern were affected by HT (Fig. 2). At the earlier time points (0.5 h and 1 h), the number of differentially regulated genes (up- and down-regulated) was higher in plants under HT than in plants under CT (Fig. 2B). This pattern was the opposite at intermediate (5 h) and late time points (11 h).

A total of 28,922 *S. tuberosum* genes were assigned to 13 phylostratigraphic groups (PS, 1–13; assigning PS1 to the most ancient genes) using a phylostratigraphic map (Fig. S1). We then computed the two transcriptomic induction indices: the iTAI and iTDI. We observed that HT strongly influences the evolutionary properties of the genes induced by *P*. *operculella* simulated herbivory. More specifically, in plants under CT, the early (0.5 h and 1 h) and late (11 h) herbivory response (up- and down-regulated) genes were predominantly younger (high iTAI) and more divergent (high iTDI), while the intermediate (5 h) response genes were predominantly ancient (lower iTAI) and more conserved (lower iTDI) genes. Contrasting patterns were observed in plants under HT: the very early (0.5 h), intermediate (5 h), and late (11 h) response genes were predominantly younger (high iTAI) and more divergent (high iTDI), while early (1 h) response genes were predominantly ancient (lower iTAI) and more conserved (lower iTDI) genes, resulting in iTAI and iTDI values going from being consistent with an hourglass pattern to being consistent with a vase pattern (Fig. 2C). Similarly, contrasting evolutionary properties of the up-regulated or down-regulated genes in HT and CT plants revealed that up-regulated genes were primarily responsible for the hourglass/vase (Fig. 2C). Taken together, temperature alter herbivory-responsive gene expression and the herbivory-responsive up- and down-regulated genes have different evolutionary properties in potato.

### Temperature influences accumulation patterns of jasmonate-associated, primary metabolism- and specialized metabolism-related genes

**Fig. 3:**
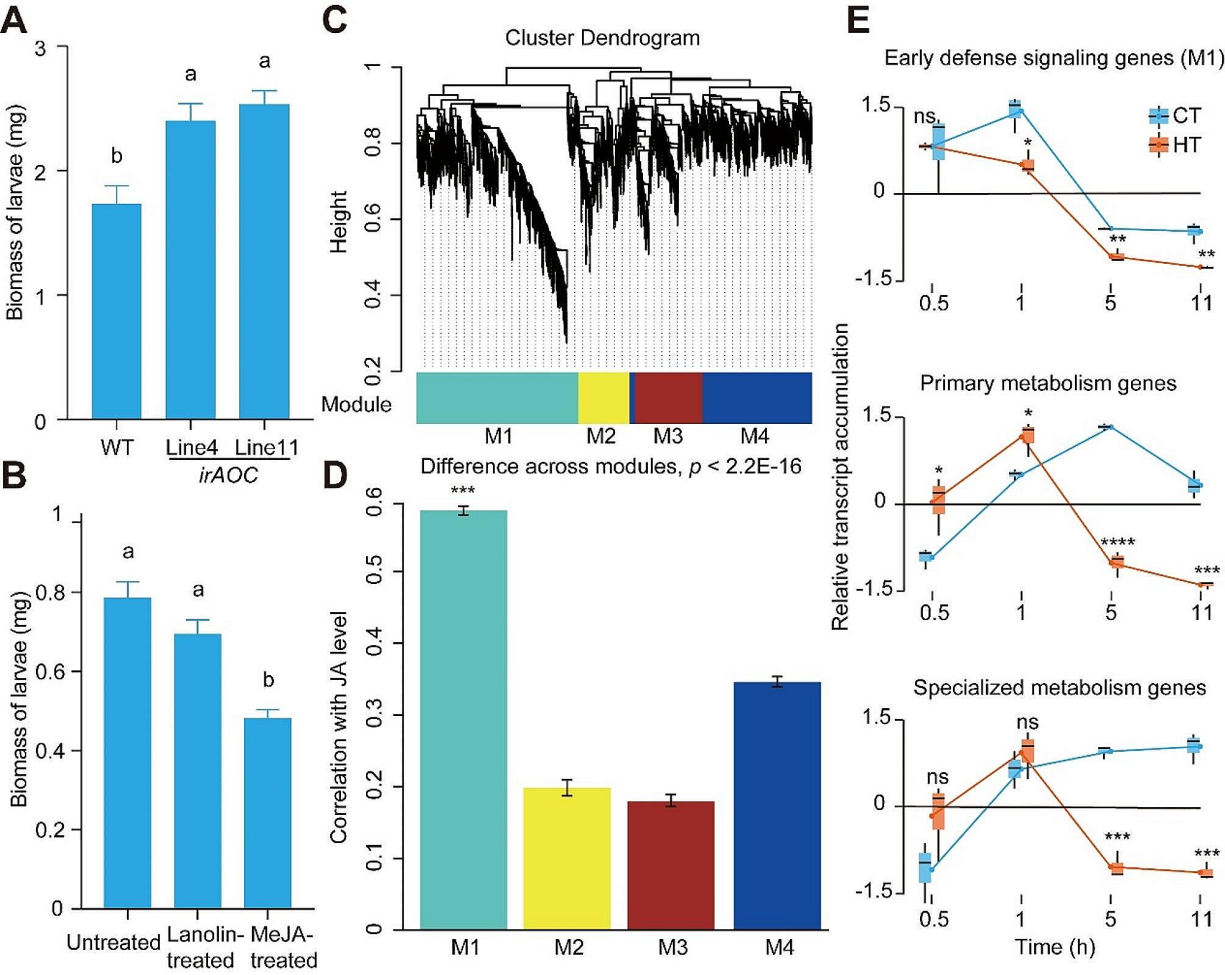
JA signaling regulates plant defense against *P. operculella*, identification of potato plants’ early defense signaling genes, and relative transcript levels of herbivory-induced early defense signaling genes (M1), primary metabolism genes and specialized metabolism genes at CT/HT. **(A)** Larval weight of *P. operculella* after six days of feeding on wild type (WT) and *irAOC* plants (*n* = 30). **(B)** Larval weight of *P. operculella* after six days of feeding on untreated, lanolin- and MeJA-treated plants (*n* = 30). Letters indicate the statistically significant differences (*P* < 0.05). Error bars indicate the mean ± SE. **(C)** The cluster dendrogram of the four modules (M1-M4). Each color indicates a different co-expression module. Y-axis indicates the height of the clustering tree. Turquoise: M1, yellow: M2, brown: M3, blue: M4. **(D)** The average correlation coefficient between each module and the herbivory-induced JA level in potato. Y-axis, the value of average correlation coefficients. Mean and SE are shown for each bar. Stars indicates a significant correlation coefficient (***, *P* < 0.001, Wilcoxon-Mann-Whitney test). **(E)** Herbivory-induced genes which showed significantly differential expression (|log_2_fold-change| ≥1 and the adjusted *P*-value < 0.05) were considered. Ns indicate no significance was found (*P* > 0.05); * indicates *P* < 0.05; ** indicates *P* < 0.01; *** indicates *P* < 0.001; **** indicates *P* < 0.0001

The JA signaling was correlated with plant defense against *P. operculella* on JA-reduced and on MeJA-treated potato plants. The detached leaf bioassays revealed that, compared to WT, *P*. *operculella* larvae accumulated more biomass on *irAOC* plants (Fig. 3A). In contrast, *P*. *operculella* larvae accumulated less biomass on MeJA-treated than untreated plants (Fig. 3B). WGCNA analysis revealed four co-expression gene modules (M1-M4) (Fig. 3C). Among them, module M1 was more correlated with herbivory-induced JA levels (Fig. 3D). A total of 1258 genes, or early jasmonate-associated genes, compose this module. A total of 2865 and 534 genes, were related to primary metabolism and specialized metabolism, respectively.

The three gene groups were found expressed in an orderly manner under CT: early jasmonate-associated gene group was highly expressed at 0.5 and 1 h, primary metabolism-related gene group at 5 h and specialized metabolism-related gene group at 11 h (Fig. 3E). Interestingly, the expression of these three groups of genes differs in plants under HT. More specifically, fewer genes were differently regulated by simulated herbivory attacked in HT plants compared to CT plants at 1, 5, and 11 h upon simulated herbivory attack. In contrast, more primary metabolism-related genes were differently regulated by simulated herbivory attacked in HT plants compared to CT plants at 0.5 and 1 h upon simulated herbivory attack. The opposite pattern was observed at 5 and 11 h upon simulated herbivory attack. The number of differentially expressed transcripts related to specialized metabolism did not differ between HT and CT plants at earlier time points but at intermediate and late time points, being more in plants under CT than under HT. Together, these results suggest that temperature alters the accumulation patterns of early jasmonate-associated defensive genes, primary metabolism-related and specialized metabolism-related genes upon simulated herbivory attack.

### Temperature modulates herbivory-induced jasmonate signaling

**Fig. 4:**
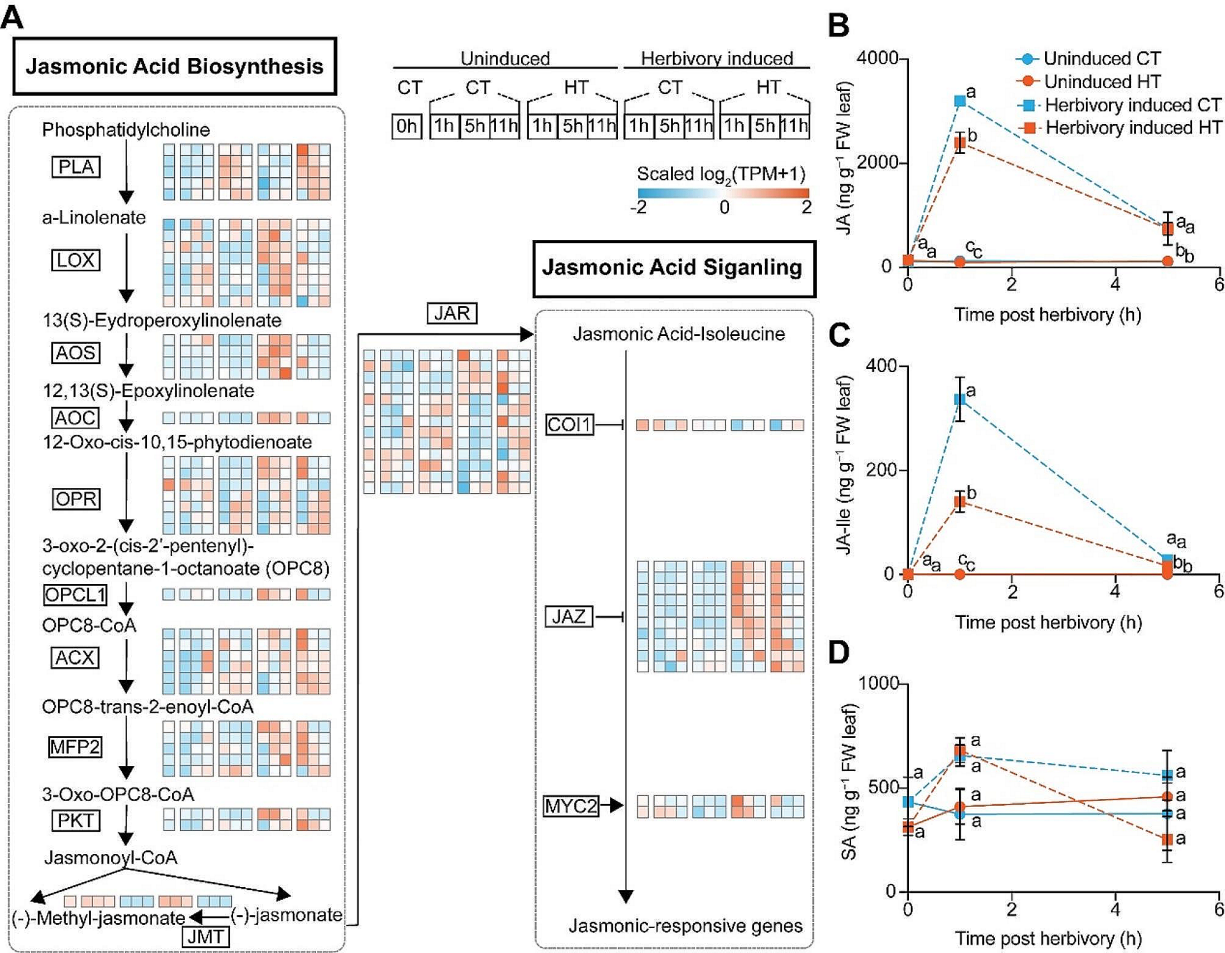
Heatmap for transcript accumulations of herbivory-induced genes in JA signaling, and phytohormone levels in potato leaves. **(A)** The JA biosynthetic and signaling pathway, and heatmaps for transcript accumulations of critical genes related to this pathway in different treatments. The levels of four phytohormones **(B)** JA, **(C)** JA-Ile, and **(D)** SA were measured in potato leaves at 0 h, 1 h, and 5 h post herbivory at CT/HT. Different line types and colors represent different treatments. Letters indicate the statistically significant differences (*P* < 0.05) at each time point. Data points indicate the mean ± SE (*n* = 4)

The herbivory-induced transcript levels of different jasmonate biosynthesis and signaling genes and jasmonate levels were affected by HT. We observed that simulated herbivory significantly induced most JA biosynthetic genes (*LOX*, *AOS*, *AOC*, *OPR*, *OPCL1*, *ACX*, *MFP2*, *PKT*) and JA signaling genes (*JAR*, *JAZ*, *MYC2*) in plants under CT (Fig. 4A). Simulated herbivory also induces these genes on plants under HT, albeit to a lesser extent than in CT plants, especially *LOX*, *AOC*, and *AOS* genes. Transcript accumulation patterns correlated well with actual jasmonate levels as HT significantly suppressed herbivory-induced JA and JA-Ile (Fig. 4B and C). Together, temperature modulates jasmonate signaling upon simulated herbivory attack.

### Temperature modulates herbivory-induced changes in primary metabolism

**Fig. 5:**
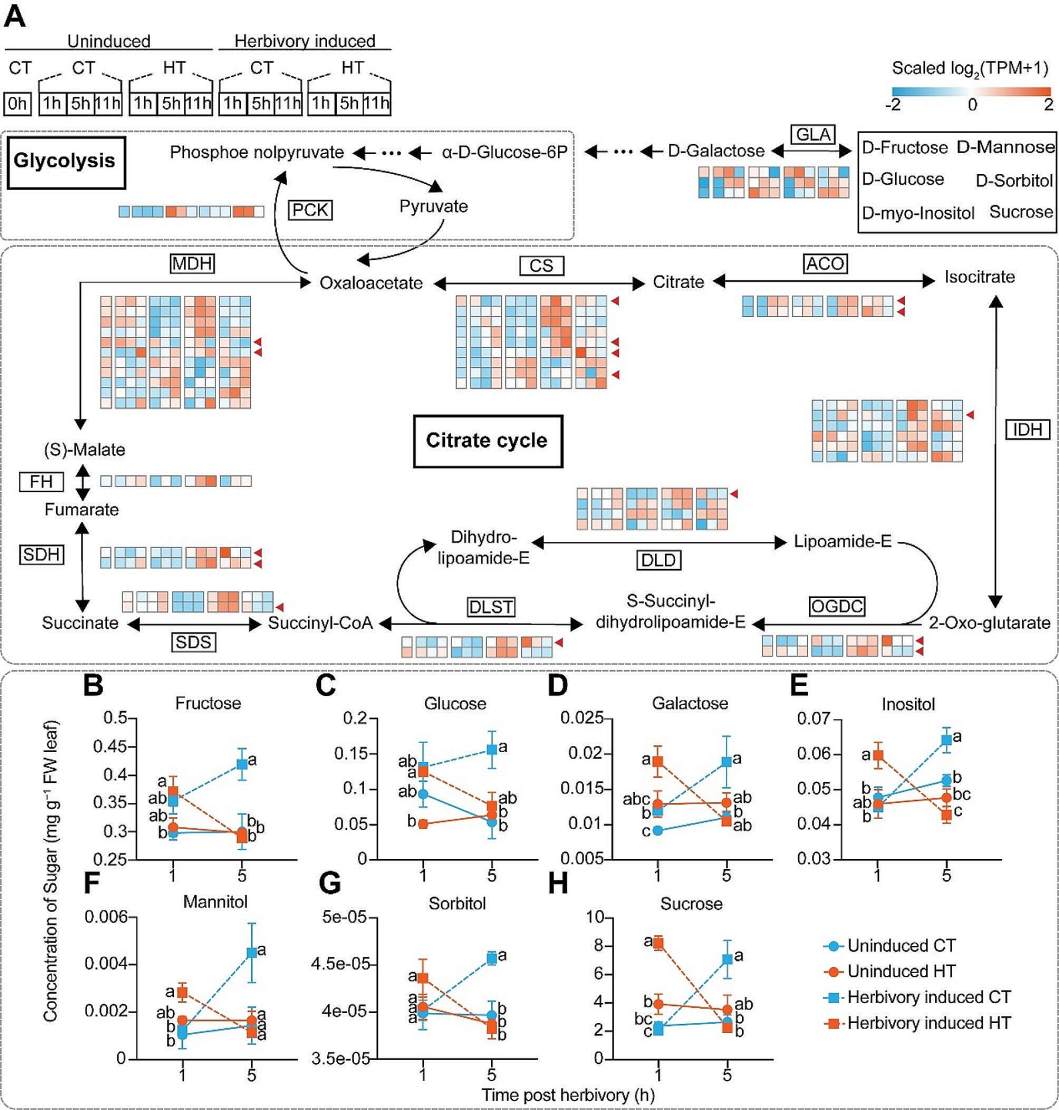
Heatmap for transcript accumulations of herbivory-induced genes related to primary metabolism, and soluble sugar levels in potato leaves. **(A)** The glycolysis and citrate cycle module, and heatmaps for transcript accumulations of critical genes related to these two pathways in different treatments. Herbivory-induced genes which are earlier-primed and suppressed by HT are marked with red triangles. **(B–H)** The concentration of seven soluble sugar (fructose, glucose, galactose, inositol, mannitol, sorbitol and sucrose) at 1 h and 5 h post herbivory at CT/HT. Different line types and colors represent different treatments. Letters indicate the statistically significant differences (*P* < 0.05) at each time point. Data points indicate the mean ± SE (*n* = 6)

The herbivory-induced transcript levels of different primary metabolism genes and primary metabolite levels were affected by HT. We observed that the transcripts of most genes of the citrate cycle (*CS*, *ACO*, *IDH*, *OGDC*, *DLD*, *DLST*, *SDS*, *SDH*, *FH*, *MDH*) were up-regulated by herbivory, and this up-regulation peaked at 5 h post herbivory (Fig. 5A). In HT conditions, while the transcripts of them were suppressed, some copies of these genes (*CS*, *ACO*, *IDH*, *OGDC*, *DLD*, *DLST*, *SDS*, *SDH*, *MDH*) were still transiently induced at 1 h post herbivory. In a similar manner, temperature also modulated the concentration of soluble sugars. More specifically, the concentration of soluble sugars (fructose, glucose, galactose, inositol, mannitol, sorbitol, sucrose) was increased upon herbivory in plants under CT, specially 5 h after simulated herbivory (Fig. 5B–H). The opposite pattern was observed in plants under HT as most of the sugars were reduced 5 h after simulated herbivory (Fig. 5B–H). Together, temperature modulates changes in primary metabolism upon simulated herbivory attack.

### Temperature modulates herbivory-induced changes in specialized metabolism

**Fig. 6:**
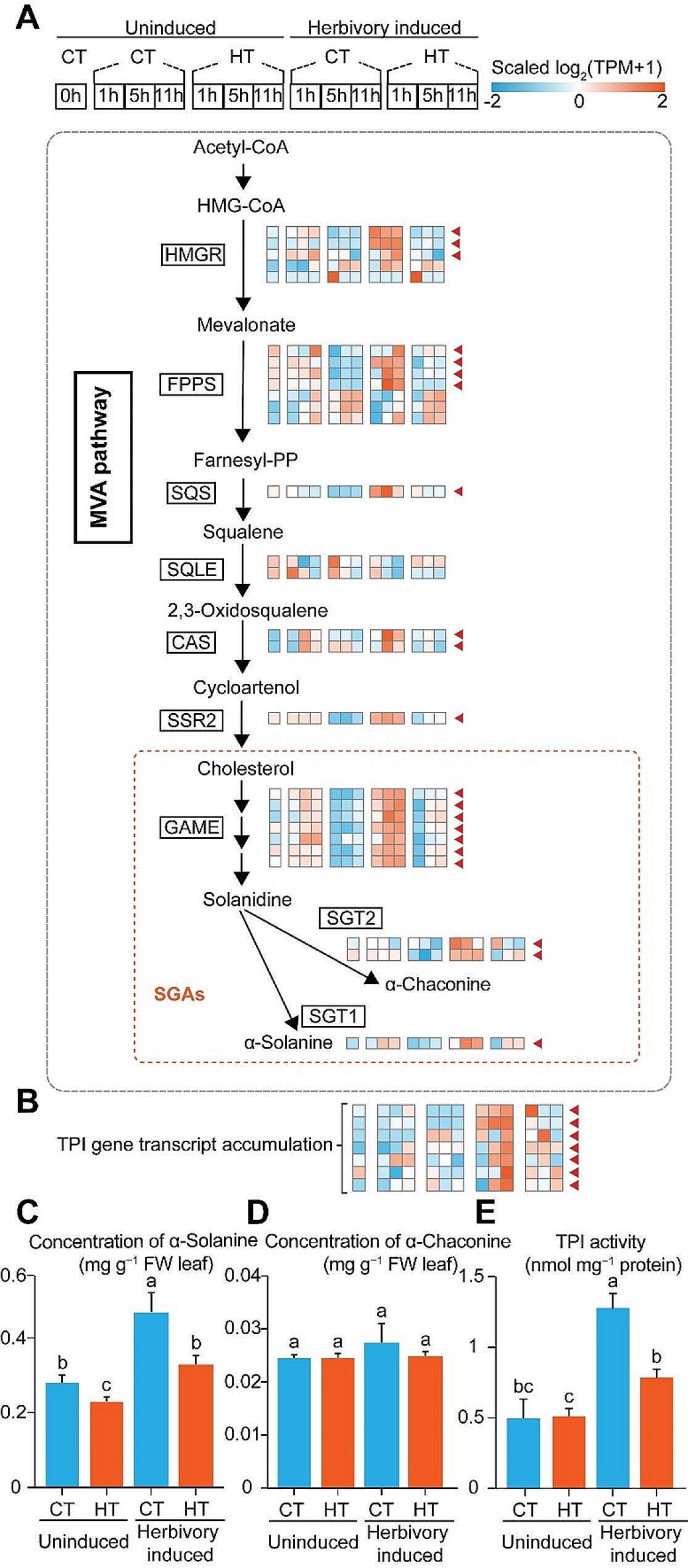
Heatmap for transcript accumulations of herbivory-induced genes related to specialized metabolism, and specialized metabolites levels in potato leaves. **(A**,** B)** The MVA pathway, and heatmaps for transcript accumulations of critical genes related to MVA pathway, SGAs and TPI in different treatments. Herbivory-induced genes which are suppressed by HT are marked with red triangles. The concentration of **(C)** α-solanine, **(D)** α-chaconine, and **(E)** TPI activity two days post herbivory at CT/HT. Letters indicate the statistically significant differences (*P* < 0.05). Error bars indicate the mean ± SE (*n* = 5)

Likewise, the herbivory-induced transcript levels of different specialized metabolism genes and specialized metabolite levels were affected by HT. In CT plants, the transcripts of most of the genes of the mevalonate (*HMGR*, *FPPS*, *SQS*, *CAS*, *SSR2*), the SGAs biosynthesis (*GAME*, *SGT1*, *SGT2*), and the TPI biosynthesis (*TPI*) pathways were up-regulated by herbivory. In contrast, the induction of these genes was weaker in HT plants (Fig. 6A and B). Accordingly, we observed that the induction of α-solanine and TPI activity was weaker in HT plants compared to CT plants (Fig. 6C and E). The content of α-chaconine was not influenced by herbivory or temperature (Fig. 6D). Hence, temperature influences the accumulation of herbivory-induced specialized metabolites in potato plants, possibly by regulating their biosynthesis.

### Jasmonate signaling contributes to the high temperature-mediated suppression of herbivory-induced defense responses

**Fig. 7:**
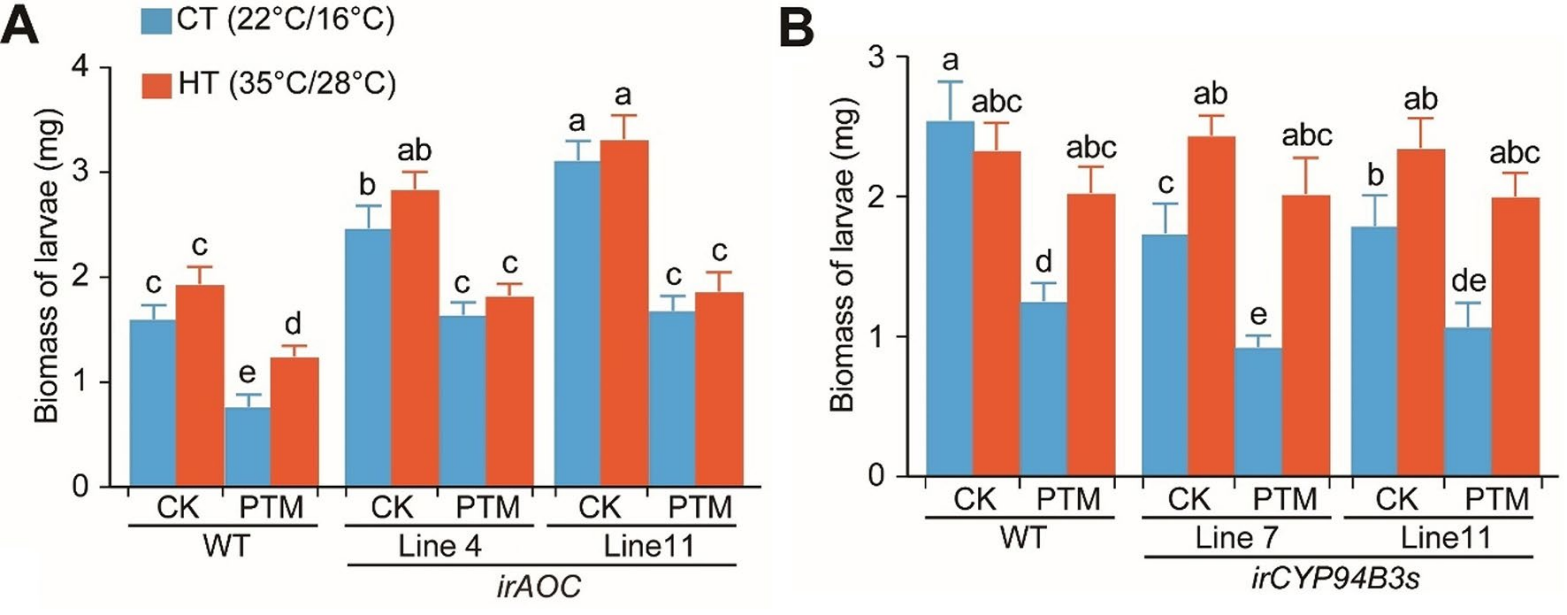
Silencing *StAOC*/*StCYP94B3s* weakened/strengthened high temperature’s suppression on potato plants’ herbivory-induced defense against *P. operculella*. **(A, B)** Larval weight of *P. operculella* after six days of feeding on WT, *irAOC* and *irCYP94B3s* leaves (*n* = 30). Plants pre-treated with either *P. operculella* simulated herbivory or none were labeled with PTM or CK. Letters indicate the statistically significant differences (*P* < 0.05). Error bars indicate the mean ± SE

A similar detached leaf bioassay was performed on WT, jasmonate-reduced (*irAOC*) and jasmonate-elevated (*irCYP94B3s*) E3-potato plants (Fig. 1A). In WT plants, insect grew similarly when fed on either undamaged leaves of plants previously exposed to HT or on undamaged leaves of plants previously exposed to CT. Interestingly, when insects fed on leaves previously subjected to simulated herbivory, they grew less compared to when insects fed on undamaged leaves, and temperature modulated these effects, similar as we observed in our first experiment (Fig. 1C). We further observed that, contrary to what was observed in WT plants, insects grew similarly when fed on leaves co-stressed by high temperature and insect herbivory with on those pre-stressed by herbivory alone, indicating temperature did not influence the performance of *P. operculella* insects on leaves previously subjected to simulated herbivory in both *irAOC* lines (Fig. 7A). Moreover, insect grew less when fed on leaves previously subjected to simulated herbivory in *irCYP94B3s* lines than in WT plants under CT. However, insect grew similarly when fed on leaves co-stressed by high temperature and insect herbivory in *irCYP94B3s* lines with WT plants, showing temperature further suppressed the reduced growth of *P. operculella* insects on leaves previously subjected to simulated herbivory in both *irCYP94B3s* lines (Fig. 7B). Collectively, these results suggest that temperature and JA act in concert to modulate plant resistance to herbivore attack.

## Discussion

The occurrence of heat waves (i.e.: transient increase in environmental temperature) has increased in recent times compared to pre-industrial times, impacting different aspects of agro-ecosystems, including crop productivity and the performance of agricultural pests [2]. The direct effect of high temperatures on herbivores has been extensively studied [72–74]. In our study, exposure to higher temperatures promoted the growth of *P*. *operculella* larvae but also increased their mortality on artificial diets (Fig. S9). However, how high temperatures in general and heatwaves, in particular, affect herbivore’s performance through plant-mediated effects remains unclear [39, 75–78]. We observed that transiently exposing potato plants to extreme temperatures significantly promoted the growth of *P*. *operculella* larvae on herbivory-induced leaves, which are cool-weather crops. This insect growth promotion observed was partially modulated by the impact of temperature on plant-induced defense induction. Moreover, considering that the temperature in real field conditions is more variable than the stable conditions we used in the lab, it would be interesting to investigate how elevated ambient temperatures in natural environments affect plants’ induced defenses against herbivores.

We observed that the induction of plant defenses in potato plants in response to *P*. *operculella* herbivory follows a phylotranscriptomic hourglass pattern consistent with iTAI and iTDI patterns from herbivory-induced transcriptome responses, similar to those observed in other plant systems such as *Nicotiana attenuata*. Moreover, the hourglass pattern was observed in bacterial-associated elicitor-induced defense signaling [27]. The analogous evolutionary hourglass might represent a general model for describing signaling cascades that are induced by different stresses. Clearly, more defense responses stimulated by different stresses need to be clarified with similar phylotranscriptomic approaches in other plant species to test the robustness of the hourglass phenomena.

**Fig. 8:**
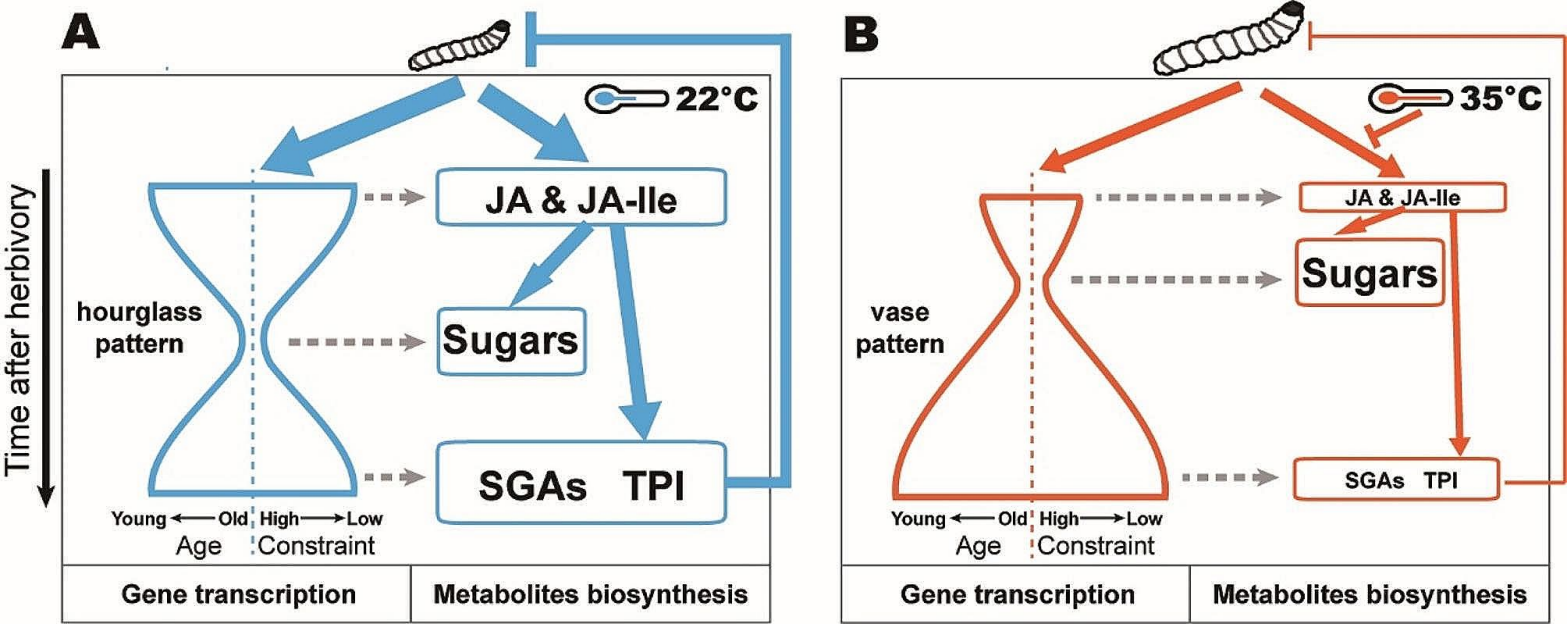
A module for the reprogramming of herbivory-induced defense responses by high temperature in potato plants. The colored lines and arrows indicate the interaction between two components. The filling amount of line and arrow represent the strength of an interaction. The font size of metabolites indicates the accumulations of compounds. **(A**,** B)** The left parts of both pictures at CT/HT represent gene transcription while the right represent metabolites biosynthesis cascade. From the module, (1) herbivory-induced defense responses can be divided into three stages: (i) herbivore attack signal perception and processing, (ii) activation of primary metabolism, (iii) activation of specialized metabolite biosynthesis, which was summarized as an evolutionary ‘hourglass’ pattern at CT, whereas HT reprogrammed this into ‘vase’ pattern; (2) the patterns were composed of calculated average herbivory-induced gene ages (iTAI profile, left) and evolutionary constraints (iTDI profile, right) at different stages; each of the profiles showed the same pattern, where gene ages reflect long-term evolutionary changes covering 4 billion years since the origin of life, and evolutionary constraints reflect short-term evolutionary changes covering 7.3**–**8 million years since the divergence of *Solanum lycopersicum*, representing ancient and recent selection pressures, respectively; (3) jasmonates (MeJA) had an impact on plants’ sugar level, SGAs and TPI accumulations (Fig. S5 and S6); (4) the biosynthesis of defense compounds at each stage were consistent with their biosynthetic gene transcript accumulation; (5) jasmonate-induced soluble sugars were still transiently induced at 1 h post herbivory although their concentration were suppressed at 5 h post herbivory at HT (Fig. 5); (6) the concentration of herbivory-induced jasmonates, SGAs, and TPI activity were suppressed at HT, which led to better *P. operculella* larvae performance on potato plants

The iTAI and iTDI presented different patterns when plants were exposed to high temperatures, which altered this phylotranscriptomic pattern from an hourglass- to a vase-shape pattern, suggesting high temperatures recruit different gene sets during the process of activation of herbivory-induced defenses. This change in phylotranscriptomic pattern was partially due to the suppression of several JA-associated, herbivory-induced genes when potato plants were exposed to high temperatures (Fig. 3E). We further performed GO enrichment analysis on herbivory-induced genes under control and high temperatures (Fig. S10). At the early stage (0.5 h and 1 h) of herbivory-induced defense process, while CT genes (early defense signaling gene group) were enriched in ‘response to endogenous stimulus’, ‘response to hormone’, and ‘kinase activity’. HT genes were enriched in ‘metabolic process’ and ‘photosynthesis’. Since the HT genes were more involved in metabolism than signaling, the phylotranscriptomic pattern under HT was predominantly ancient (lower iTAI) and more conserved (lower iTDI) at 0.5 h and 1 h. Similarly, CT genes (primary and specialized metabolism gene groups) at the intermediate (5 h) and late (11 h) stages of herbivory-induced defense process had different GO terms compared with HT genes. Since the HT genes were less enriched in metabolic process than CT genes, the phylotranscriptomic pattern under HT was predominantly younger (high iTAI) and more divergent (high iTDI) at 5 h and 11 h. These results suggested that HT’s differential selection of the three plant’s defense gene groups at each stage of herbivory-induced defense process led to different gene group functions form CT’s, resulting in a different phylotranscriptomic pattern (Fig. 8). An alternative hypothesis is several plant genes associated with heat stress response, which contain large numbers of ancient and conserved genes [79], were activated by high temperature exposure, especially in the early stage (0.5 h and 1 h) post the herbivory, and revealing how high temperature influence all stages of herbivory-induced gene activation in future will promote the identification of key regulators in this complex signaling cascade.

Plant’s herbivory-induced defense responses are fined-tuned by phytohormonal signaling cascades in *Planta* [80–82]. Among them, jasmonates are master regulators of several herbivory-induced defense responses [16, 83, 84]. We observed that JA, JA-biosynthesis and JA-signaling genes, are strongly induced upon herbivory in potato plants. Alterations in herbivory-induced JA were usually accompanied by changed accumulation of primary and specialized metabolites in many plants [23, 85–88]. In our study, the primary (soluble sugars) and secondary (SGAs, TPI) metabolites, together with their biosynthetic genes, are strongly induced upon herbivory. Meanwhile, levels of these metabolites and their related biosynthetic genes were strongly activated by methyl jasmonate (MeJA) treatment (Fig. S5 and S6), indicating that JA-signaling also plays an important role in potato’s induced defense against the *P*. *operculella* (Fig. 8). Indeed, suppressing herbivory-induced JA accumulation in JA-reduced plants significantly weakened their defense against *P*. *operculella*, whereas enhancing herbivory-induced JA-Ile accumulation in JA-Ile-elevated plants and applying MeJA both strengthened their defense against *P*. *operculella*. We demonstrated that JA signaling plays an important role in plants’ defense against *P*. *operculella*. Previous studies indicated that JA signaling is also related with HT [89, 90]. However, the co-stress-induced pattern by herbivory and high temperature remained unclear.

In our study, high temperature suppressed the herbivory-induced accumulation of jasmonates, primary and specialized metabolites in potato plants. The decrease in soluble sugar level and transcript accumulation is possibly attributed to a reduction in photosynthesis parameters due to stomata closure under elevated temperature [91]. Also, our previous research show that the emission of the volatile organic compounds (VOCs) in potato (cv. Lishu 6) leaves were enhanced by high temperature treatment, which subsequently affected the plants’ attractiveness to the *P*. *operculella* and its egg parasitoid wasp [39]. This distinction might be due to the use of different cultivars, whilst it is also possible that plants may differentially regulate the production of volatile and nonvolatile secondary compounds under elevated temperature. While the alteration of levels of herbivory-induced secondary metabolites (i.e.: polyphenol oxidase and glucosinolates) by high temperature have been found in many plants including tomato and watermelon [92, 93], evidence for the suppression of herbivory-induced primary metabolism by high temperature remains scarce. We propose that this suppression is related to the JA-signaling in potato plants, since the herbivory-induced JA biosynthetic genes and JA accumulations were suppressed by high temperature. However, whether suppressing JA-signaling could benefit the plant response to high temperature remains to be clarified in future.

To gain insight into the suppression of JA-regulated defense responses associated with increased *P*. *operculella* larval weight gain under high temperature, we used JA-reduced *irAOC* and JA-Ile-elevated *irCYP94B3s* mutants in insect detached leaf bioassays to investigate the contribution of JA signaling in this context. Firstly, we observed that *P*. *operculella* larvae reared on WT leaves previously subjected to insect herbivory accumulated more biomass under high temperature than control temperature. However, this difference between the two temperature regimes was eliminated when the larvae were grown on herbivory-induced detached JA-reduced leaves. Secondly, unlike the reduced biomass on JA-Ile-elevated plants under control temperature, *P. operculella* larvae accumulated similar biomass on JA-Ile-elevated plants with WT plants under high temperature. We concluded that high temperature diminished plants’ herbivory-induced defense and JA signaling participated in this regulation [94]; thus leading to higher biomass of *P*. *operculella* under high temperature. Similar results were reported in recent studies that the defense mechanism of plants against insect pests is diminished by climate change in aspects of warming-mediated host-plant quality such as poor-nutrient foliage, decreased defensive compounds, and increased sensitivity to attacks [95–98]. Future research efforts should focus on strategies to enhance crop defenses against herbivores in warming ecosystems.

In summary, our study shows that transient exposure to high temperature decreases JA signaling mediated plant-induced resistance to herbivore attack by reprogramming plant transcripts and metabolome (Fig. 8). According to previous studies in other plant-insect interacting systems, positive effects of elevated ambient temperature on plants’ herbivore defense were also observed [5, 99–101]. Potato is a cool-weather crop whose physiology is strongly affected by high temperature [102, 103]. It is likely that a ‘resource reallocation’ happened in potato plants when high temperature and herbivory stresses coexisted. In plants that are more adapted to eco-niche with warmer temperature, a different pattern may occur for their defense signaling activation, resource allocation and defensive secondary compounds production up on herbivory.

## Conclusions

In this study, we found that transient exposure to high temperature constrains JA signaling-induced defenses in potato plants against the attacks of *P*. *operculella* using two potato varieties. These effects are accompanied by changes in phylotranscriptomic patterns of herbivory-responsive genes, accumulation patterns of the early defense signaling molecule jasmonate-associated, primary metabolism- and specialized metabolism-related genes, and by changes in the production of primary and specialized metabolites. The two varieties showed similar patterns in detached leaf bioassay, transcript and phytohormone accumulation upon 1 h herbivory.

Thus, our study provides mechanistic explanations of how temperature reprogram plant defense against herbivores and insight into how the outcome of crop-herbivore interactions can be affected by global climate change.

### Electronic supplementary material

Below is the link to the electronic supplementary material.

Supplementary Material 1

Supplementary Material 2

Supplementary Material 3

## Data Availability

All RNA-Seq reads data have been deposited in Short Read Archive (SRA) at the NCBI database (Accession: PRJNA903553).

## Abbreviations

ABA: Abscisic acid
ANOVA: One-way analysis of variance
AOC: Allene oxide cyclase
CT: Control temperature
HT: High temperature
iTAI: Induced transcriptome age index
iTDI: Induced transcriptome divergence index
JA: Jasmonic acid
JA-Ile: JA-isoleucine
MeJA: Methyl jasmonate
MEs: Module eigengenes
OS: Oral secretions
PS: Phylostrata
qRT-PCR: Quantitative real-time PCR
SA: Salicylic acid
SGAs: Steroidal glycoalkaloids
TPI: Trypsin proteinase inhibitor
TPM: Transcript per million
VOC: Volatile organic compound
WGCNA: Weighted gene co-expression network analysis
