# Supplementary material for: Heat stress reprograms herbivory-induced defense responses in potato plants
Source: BMC Plant Biol. 2024 Jul 17;24:677. doi: 10.1186/s12870-024-05404-x (PMC11253553; doi:10.1186/s12870-024-05404-x)
Supplement: Supplementary file 1 — Supplementary Material 1 [file 12870_2024_5404_MOESM1_ESM.docx]

## Supporting information

Article title: **Heat stress reprograms herbivory-induced defense responses in potato plants**

Authors: Jian Zhong, Jinyi Zhang, Yadong Zhang, Yang Ge, Wenjing He, Chengjuan Liang, Yulin Gao, Zengrong Zhu, Ricardo A. R. Machado and Wenwu Zhou

**Fig. S1** Phylostratigraphy map of *Solanum tuberosum* genes.

**Fig. S2** Construction and validation of *StAOC* RNA interference vectors and transgenic regenerated potato plants.

**Fig. S3** The majority of early jasmonate-associated genes were induced by 1 h herbivory in both RH and E3 potato cultivars.

**Fig. S4** Gene Silencing efficiency and growth of *irAOC* potato plants.

**Fig. S5** Heatmap for transcript accumulations of primary metabolism-related genes, and soluble sugar levels in potato leaves treated with MeJA.

**Fig. S6** Heatmap for transcript accumulations of specialized metabolism-related genes, and specialized metabolites levels in potato leaves treated with MeJA.

**Fig. S7** TPI activity in potato leaves with and without herbivory.

**Fig. S8** Heatmap comparison of seven selected genes’ transcript accumulations quantified in RNA-Seq and qRT-PCR.

**Fig. S9** *P. operculella* larvae bioassays on artificial diets at CT/HT.

**Fig. S10** The Gene Ontology (GO) terms enriched in leaves co-stressed with high temperature and insect herbivory (HT) and stressed with herbivory alone (CT) genes in potato plants.

**Table S1** Primer information for gene clone, RNA interference and qRT-PCR.

**Table S2** Annotation from protein coding gene sequences in *Solanum tuberosum*.

**
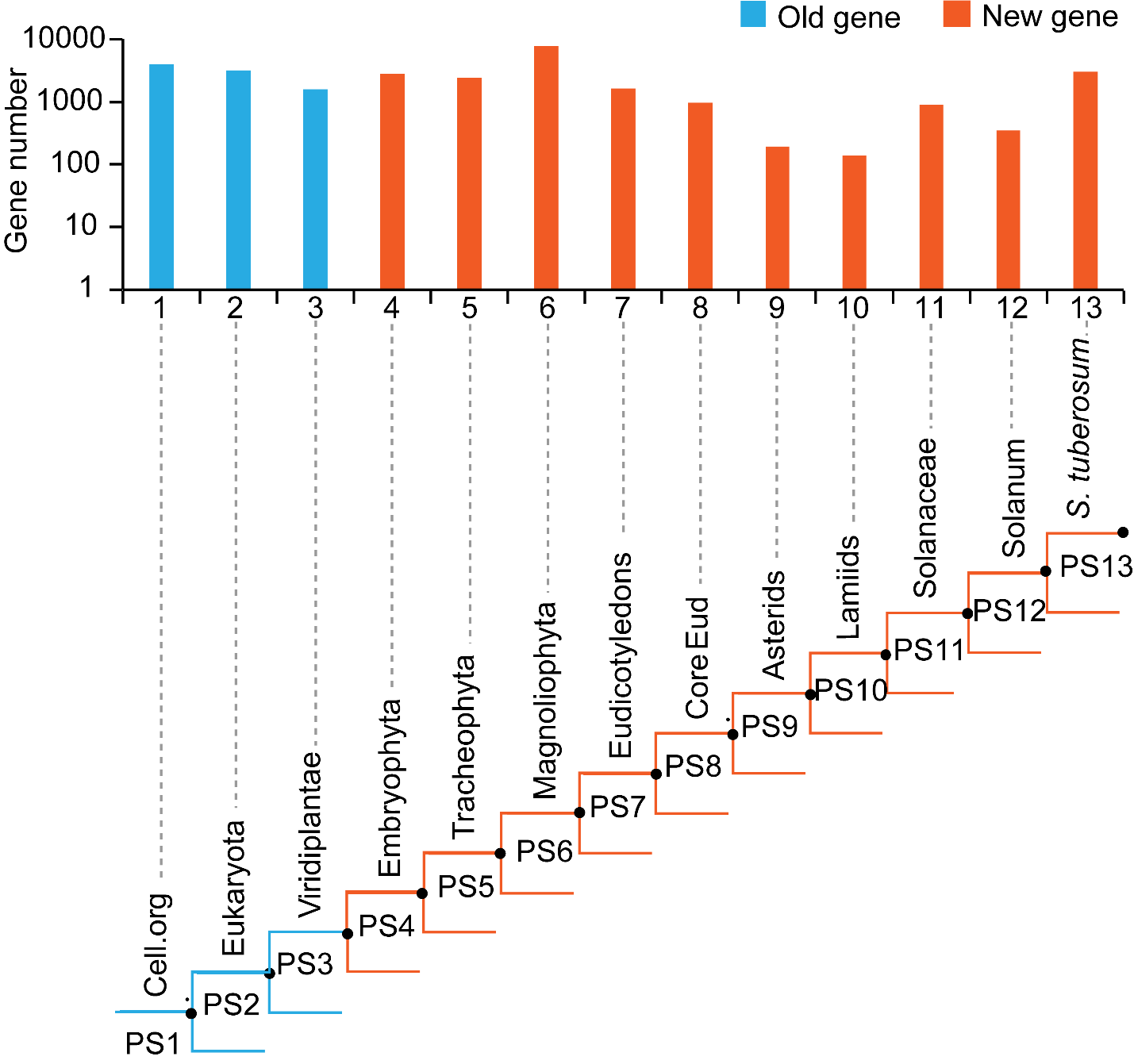
**

**Fig. S1** Phylostratigraphy map of *Solanum tuberosum* genes. Each gene was assigned to a phylostratigraphic (PS) group and the phylostratigraphic map was created by dividing the evolutionary history of *S. tuberosum* into 13 separate phylostratigraphic groups.


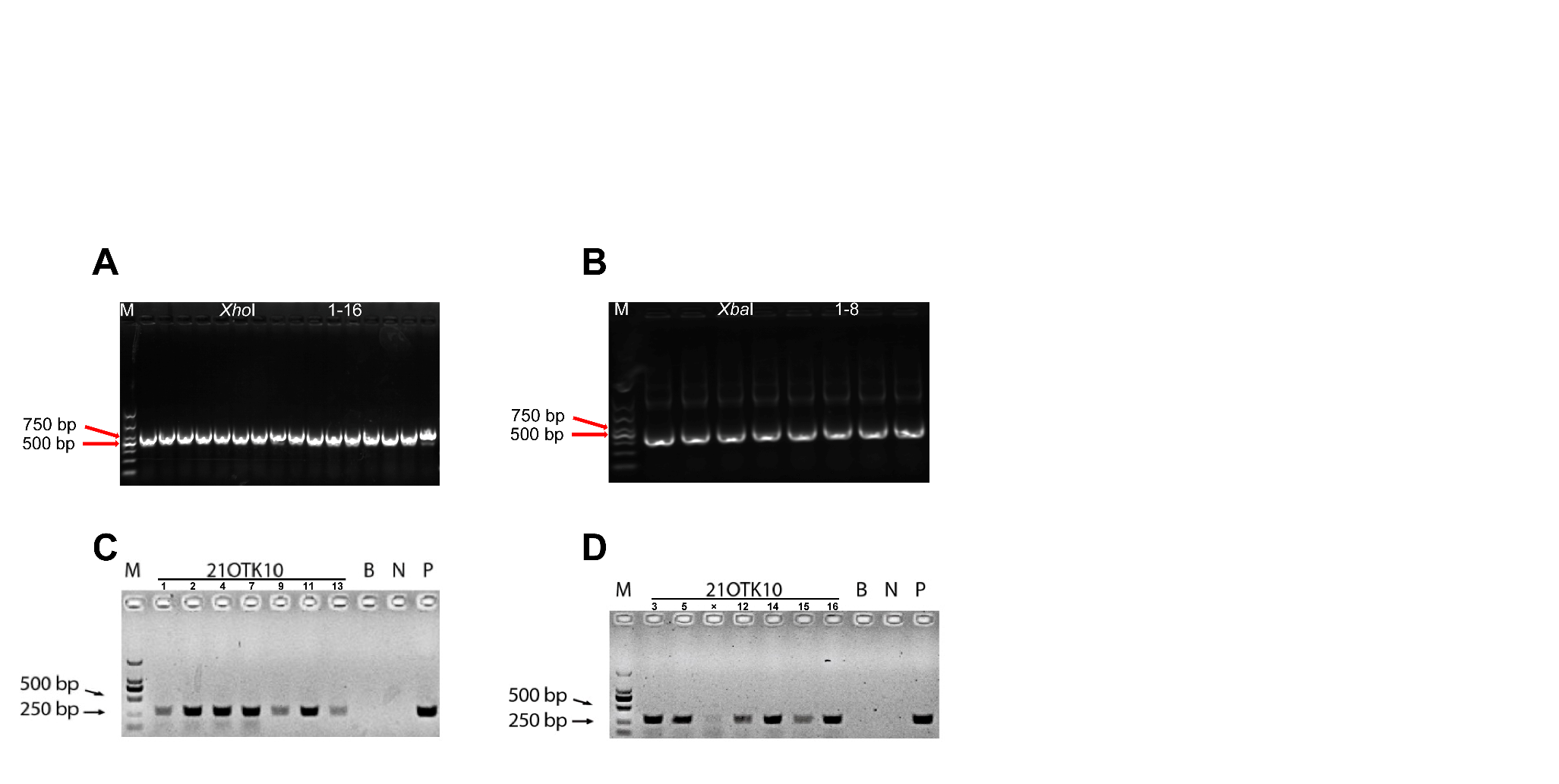


**Fig. S2** Construction and validation of *StAOC* RNA interference vectors and transgenic regenerated potato plants. Validation of colony PCR for recombination reactions with primers at both ends of *Xho*I (**A**, n=16) and *Xba*I (**B**, n=8). Validation of PCR on 14 transformed plants and 13 positive transformed plants with strain numbers 1, 2, 4, 7, 9, 11, 13 **(C)**, 3, 5, 12, 14, 15, 16 **(D)**. 21OTK09 was the production number of the transformed plant. (M: marker; B: blank control; N: negative control; P: positive control)


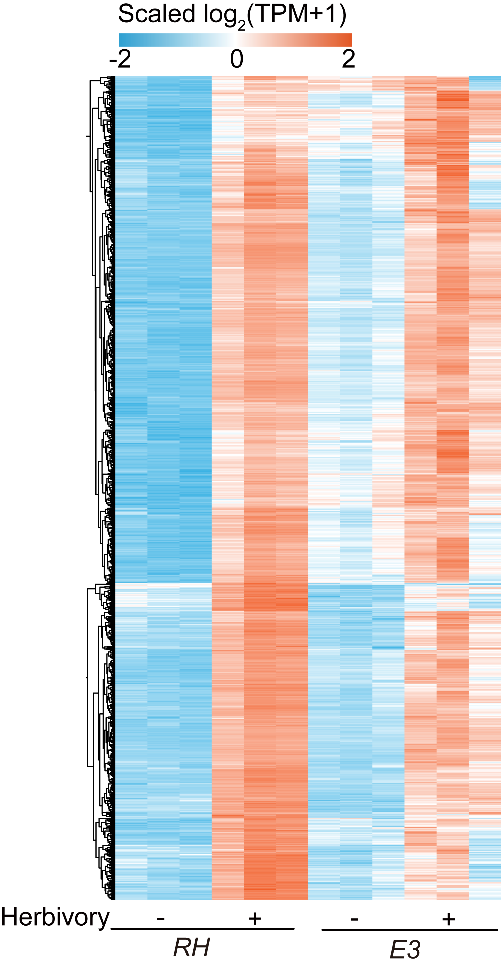


**Fig. S3** The majority of early jasmonate-associated genes were induced by 1 h herbivory in both RH and E3 potato cultivars. The patterns of transcript levels of jasmonate-associated gene group upon herbivory were similar between RH and E3 cultivars.


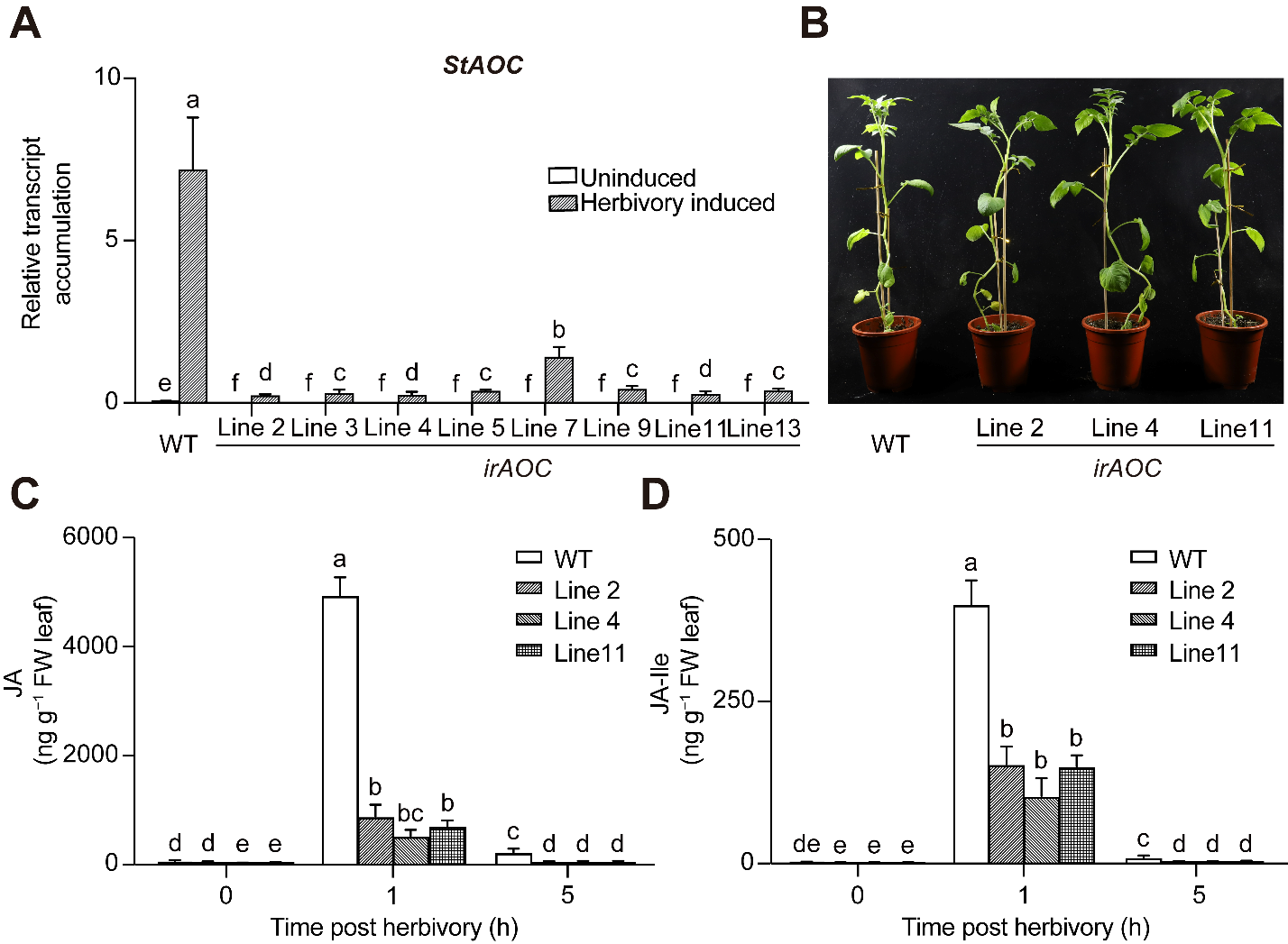
**Fig. S4** Gene Silencing efficiency and growth of *irAOC* potato plants. **(A)** Transcript accumulations of *StAOC* in herbivory-induced and uninduced potato leaves in WT and eight JA-deficient lines (*irAOC*-Line 2, Line 3, Line 4, Line 5, Line 7, Line 9, Line11 and Line13, n=3) at 1 h. **(B)** Growth of WT and three JA-deficient lines (*irAOC*-Line 2, Line 4 and Line11). **(C)** JA and **(D)** JA-Ile levels were measured in WT and JA-deficient potato plants’ leaves at 0 h, 1 h, and 5 h post herbivory. Different letters on the top of the columns indicate differences at *P* < 0.05. Error bars represent the mean ± standard error (SE) for each.

**
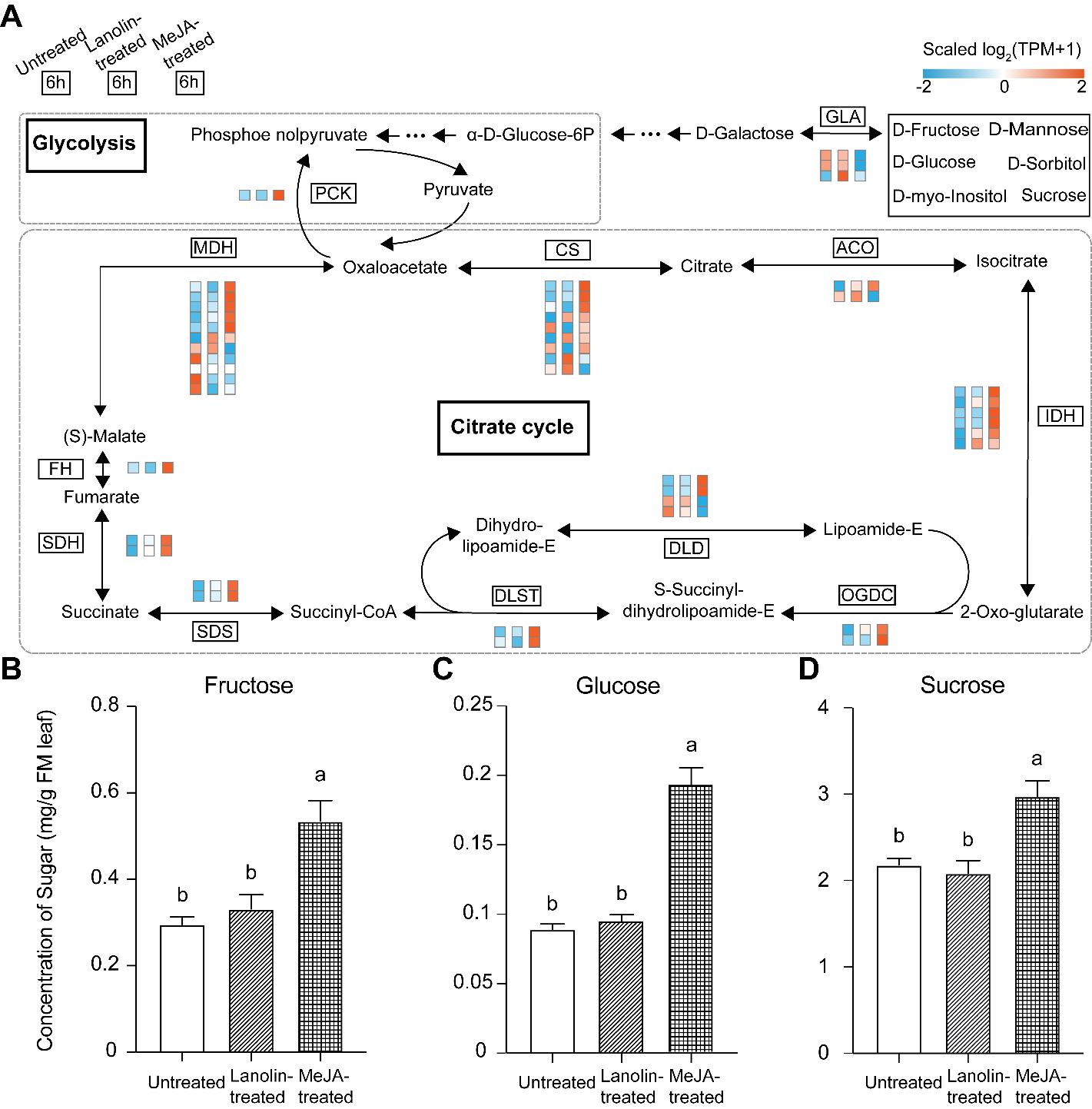
**

**Fig. S5** Heatmap for transcript accumulations of primary metabolism-related genes, and soluble sugar levels in potato leaves treated with MeJA. **(A)** The glycolysis and citrate cycle module, and heatmaps for transcript accumulations of critical genes related to these two pathways under methyl jasmonate (MeJA) treatment. **(B­–D)** The concentration of three soluble sugar (fructose, glucose and sucrose) at 6 h post herbivory. Letters indicate the statistically significant differences (*P* < 0.05). Error bars indicate the mean ± SE (n=6).


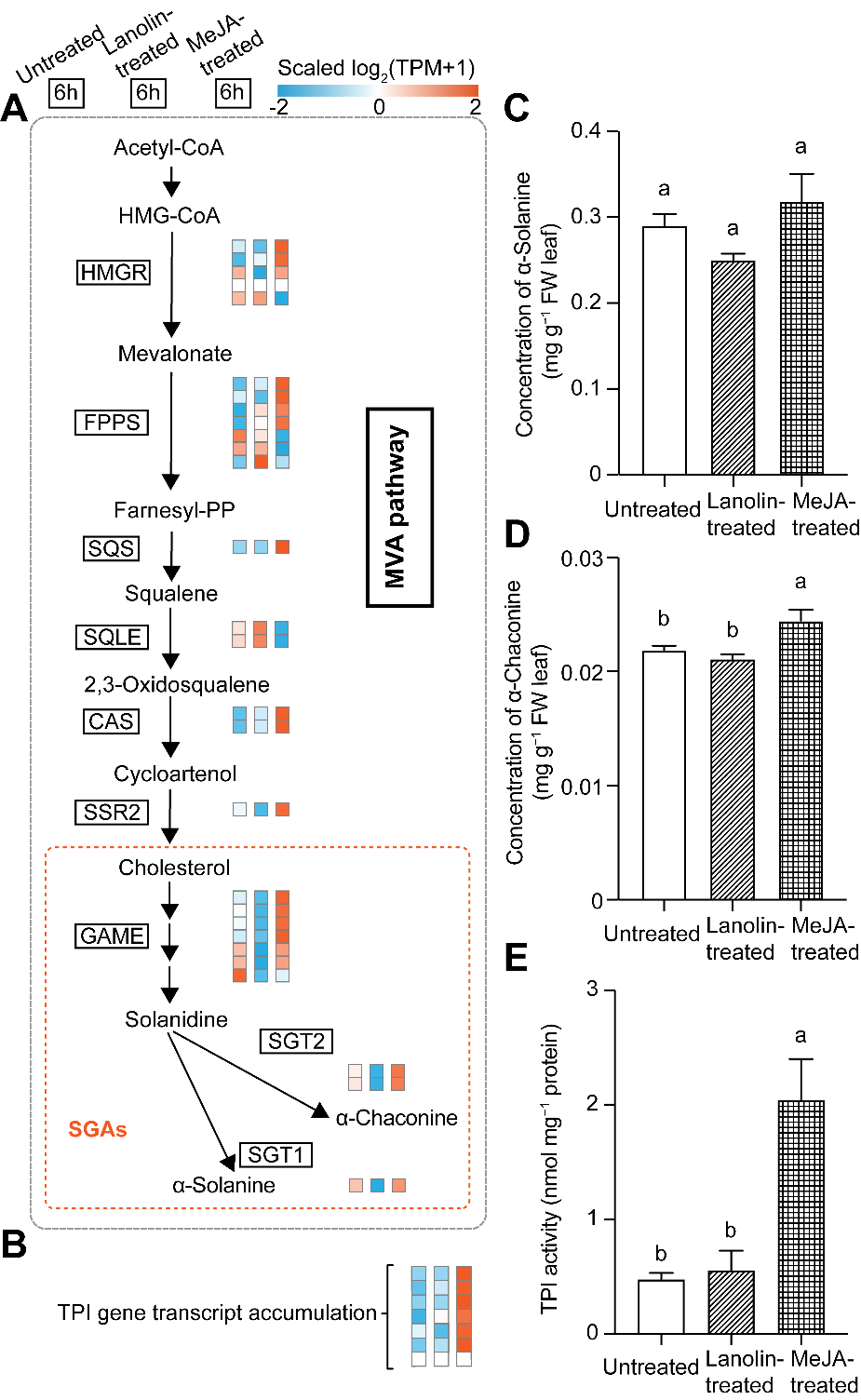


**Fig. S6** Heatmap for transcript accumulations of specialized metabolism-related genes, and specialized metabolites levels in potato leaves treated with MeJA. **(A, B)** The MVA pathway, and heatmaps for transcript accumulations of critical genes related to MVA pathway, SGAs and TPI under methyl jasmonate (MeJA) treatment. The concentration of **(C)** α-solanine, **(D)** α-chaconine, and **(E)** TPI activity two days post herbivory. Letters indicate the statistically significant differences (*P* < 0.05). Error bars indicate the mean ± SE (n=5).


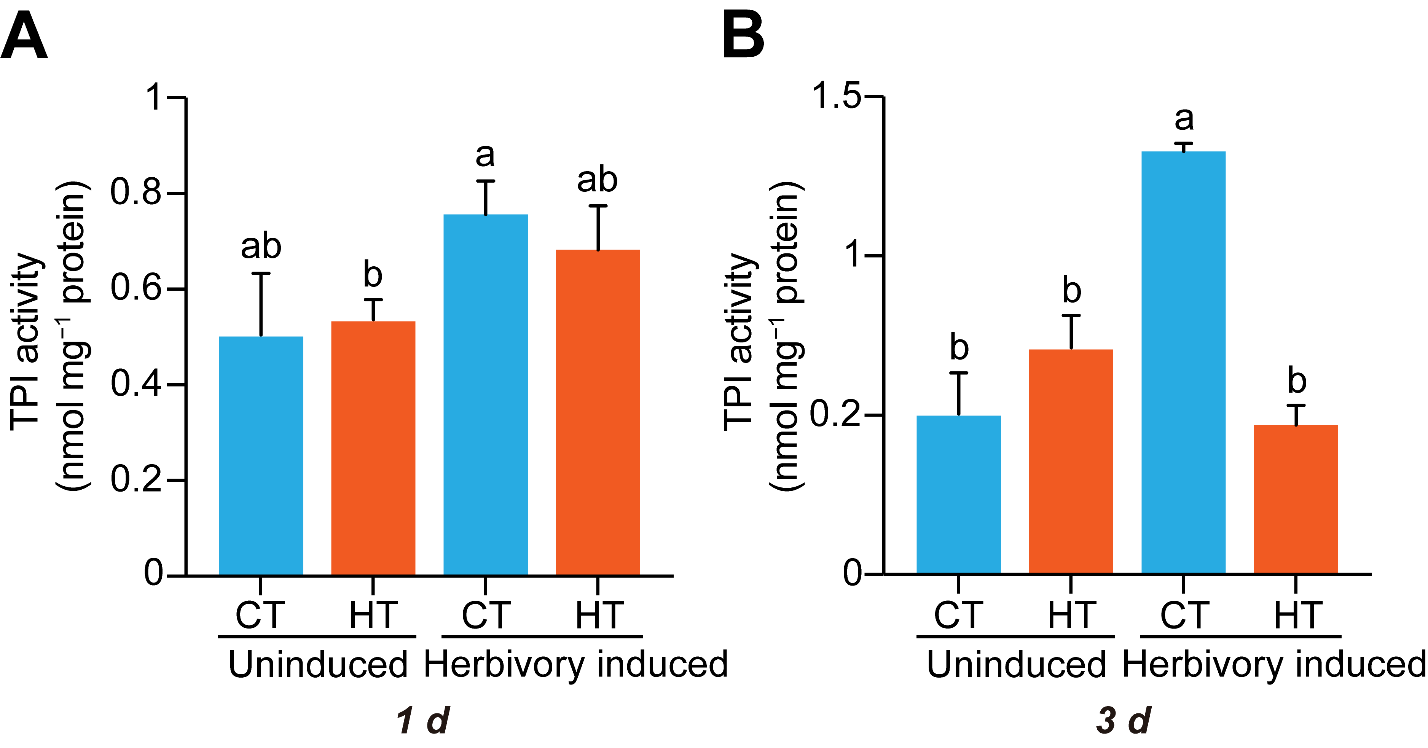


**Fig. S7** TPI activity in potato leaves with and without herbivory. **(A)** TPI activity was measured one day post herbivory at CT/HT. **(B)** TPI activity was measured three days post herbivory at CT/HT. Different letters on the top of the columns indicate differences at *P* < 0.05. Error bars represent the mean ± standard error (SE) for each.


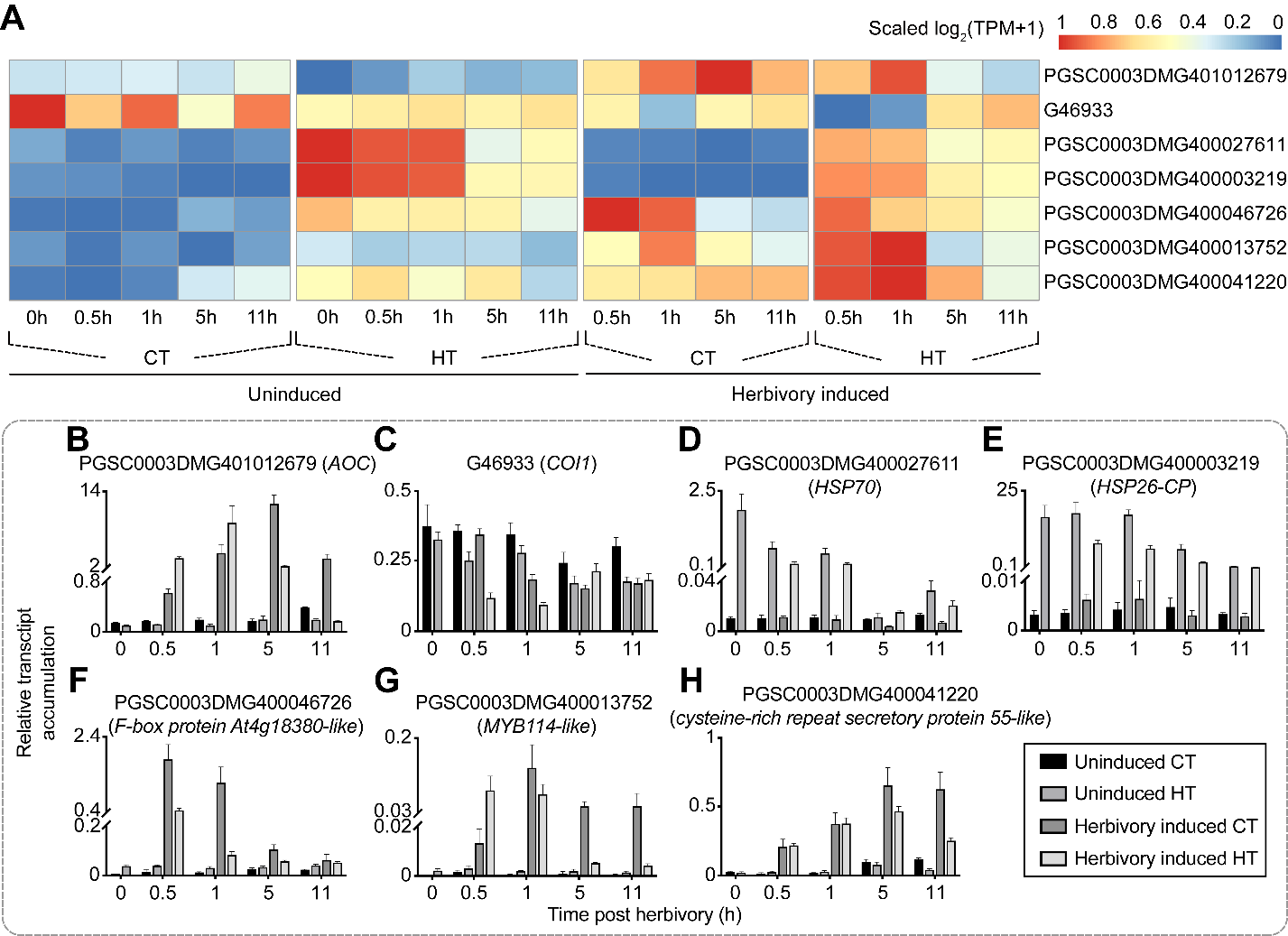


**Fig. S8** Heatmap comparison of seven selected genes’ transcript accumulations quantified in RNA-Seq and qRT-PCR. **(A)** Heatmap comparison of seven selected genes’ (two module ‘M1’ genes, two HT-induced genes, three herbivory-induced genes) transcript accumulations via RNA-Seq. **(B­–H)** Their transcript accumulations were verified via qRT-PCR.


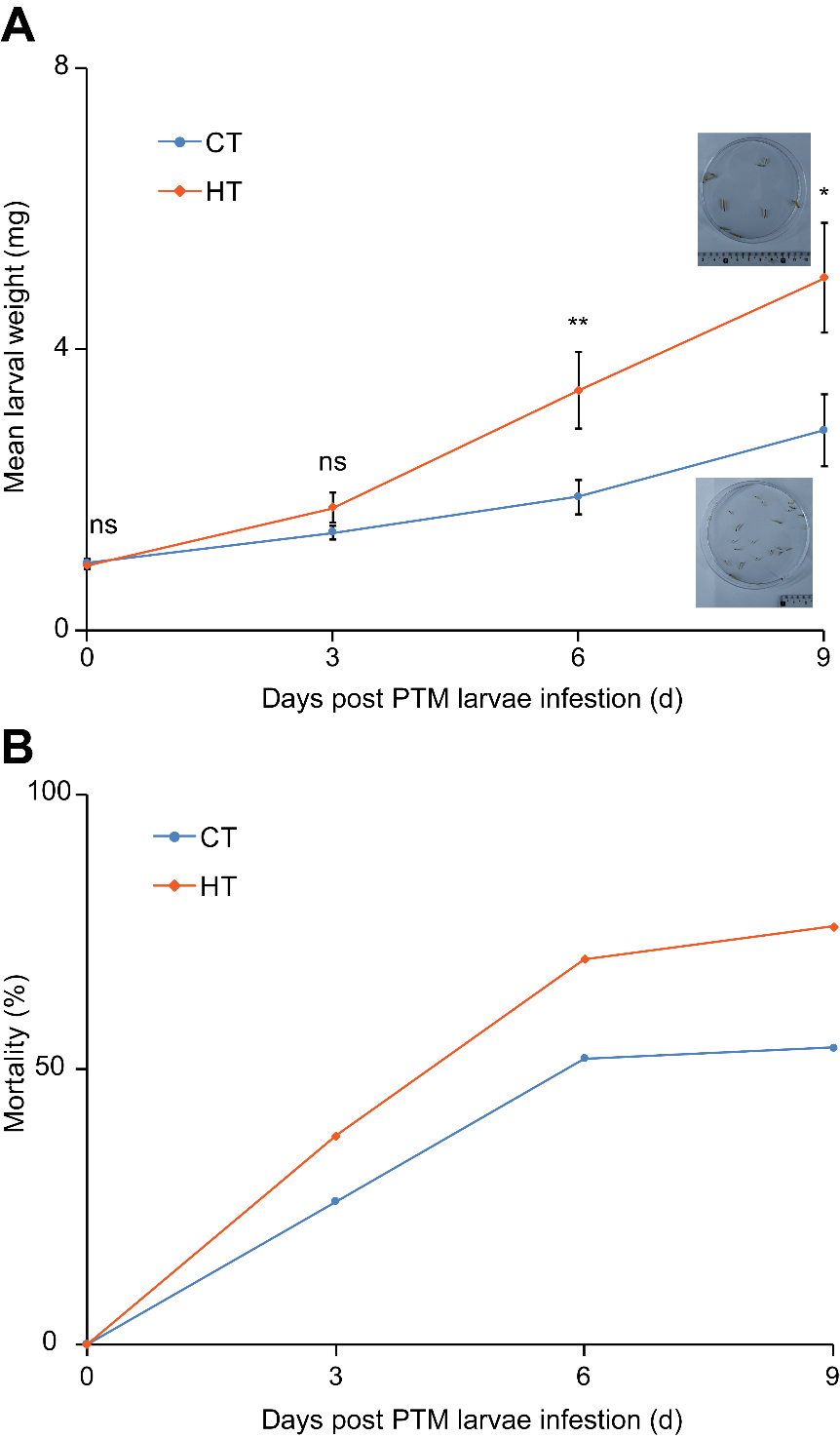


**Fig. S9** *P. operculella* larvae bioassays on artificial diets at CT/HT. The hatched larvae were first fed on potato tubers until they reached the 2nd-instar stage. After this, the larvae were starved for 6 h and then fed on an artificial diet for each. Artificial diets were changed every 3 days. **(A)** Larval weights and **(B)** mortality of larvae on artificial diets at control or high temperature; (22/16 or 35/28 ℃, day/night n=50) were measured. Different line colors represent different treatments. Asterisks indicate the statistically significant differences (*P* < 0.05) at each time point. Data points indicate the mean ± SE.


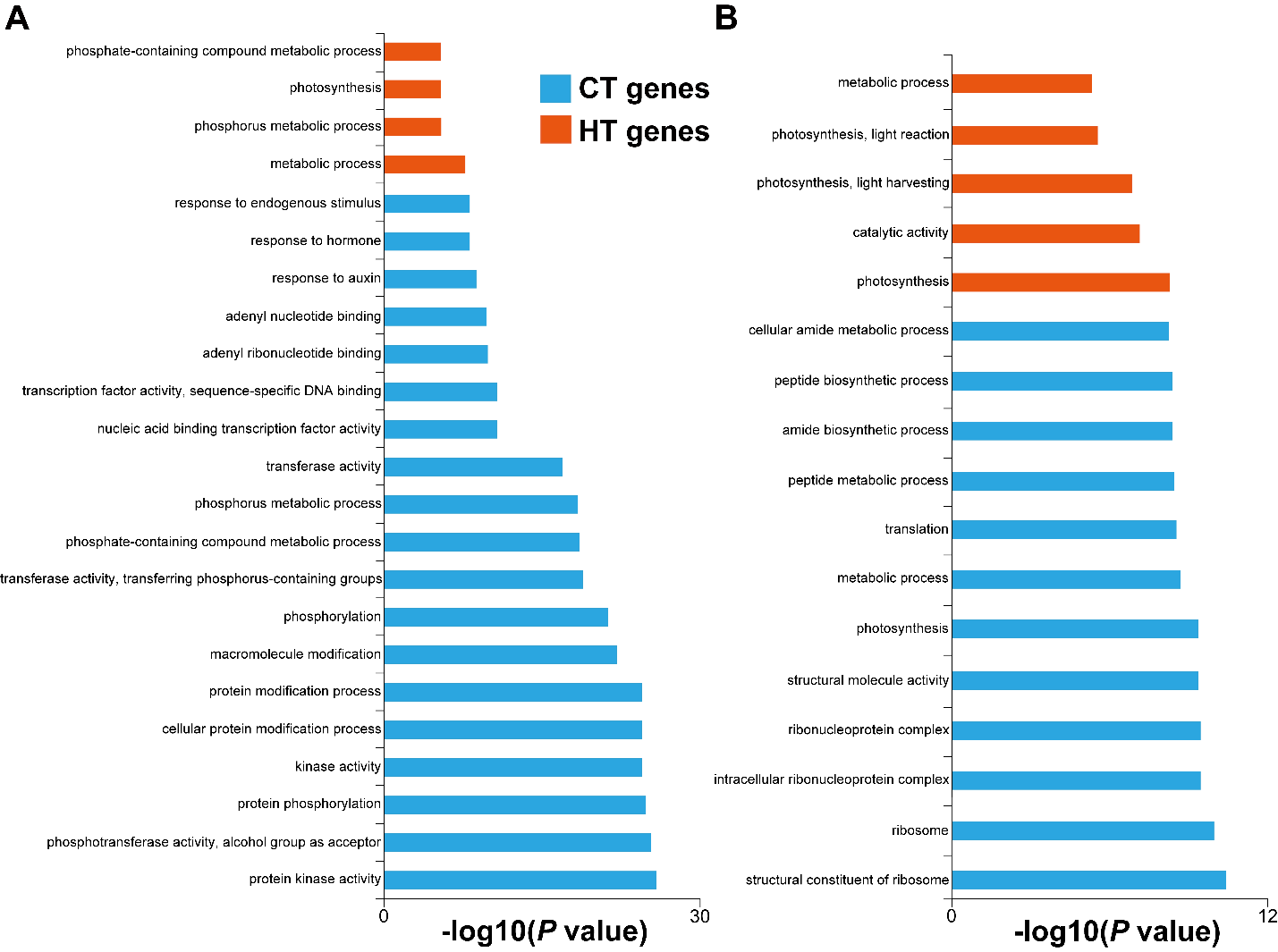


**Fig. S10** The Gene Ontology (GO) terms enriched in leaves co-stressed with high temperature and insect herbivory (HT) and stressed with herbivory alone (CT) genes in potato plants. GO terms of CT and HT genes were analyzed at **(A)** 0.5 h and 1 h, and **(B)** 5 h and 11 h post herbivory. The CT and HT genes are shown in different colors.
